# A multi-axis robot-based bioprinting system supporting natural cell function preservation and cardiac tissue fabrication

**DOI:** 10.1016/j.bioactmat.2022.02.009

**Published:** 2022-02-19

**Authors:** Zeyu Zhang, Chenming Wu, Chengkai Dai, Qingqing Shi, Guoxin Fang, Dongfang Xie, Xiangjie Zhao, Yong-Jin Liu, Charlie C.L. Wang, Xiu-Jie Wang

**Affiliations:** a Institute of Genetics and Developmental Biology, Innovation Academy of Seed Design, Chinese Academy of Sciences, Beijing, 100101, China; b Beijing National Research Center for Information Science and Technology, Department of Computer Science and Technology, Tsinghua University, Beijing, 100084, China; c Department of Mechanical, Aerospace and Civil Engineering, The University of Manchester, Manchester, M20 4BX, UK; d Faculty of Industrial Design Engineering, Delft University of Technology, Delft, 2628, the Netherlands; e University of Chinese Academy of Sciences, Beijing, 100049, China

**Keywords:** Six degree-of-freedom robot, 3D bioprinting, Artificial organ engineering, Print-and-culture, Cardiac tissue fabrication

## Abstract

Despite the recent advances in artificial tissue and organ engineering, how to generate large size viable and functional complex organs still remains as a grand challenge for regenerative medicine. Three-dimensional bioprinting has demonstrated its advantages as one of the major methods in fabricating simple tissues, yet it still faces difficulties to generate vasculatures and preserve cell functions in complex organ production. Here, we overcome the limitations of conventional bioprinting systems by converting a six degree-of-freedom robotic arm into a bioprinter, therefore enables cell printing on 3D complex-shaped vascular scaffolds from all directions. We also developed an oil bath-based cell printing method to better preserve cell natural functions after printing. Together with a self-designed bioreactor and a repeated print-and-culture strategy, our bioprinting system is capable to generate vascularized, contractible, and long-term survived cardiac tissues. Such bioprinting strategy mimics the *in vivo* organ development process and presents a promising solution for *in vitro* fabrication of complex organs.

## Highlights

• Developed a six-axis robot arm-based bioprinter to enable all directional cell printing by single- or multi-robot operation.
• Applied a hydrophobic force-based cell attachment approach to integrate printed cells with complex-shaped vascular scaffolds.
• Designed a repeated print-and-culture strategy to mimic the *in vivo* organ development process.
• Achieved vasculogenesis and angiogenesis of bioprinted blood vessels and long-term survival of bioprinted cardiac tissues.

## Graphical abstract

**Figure:**
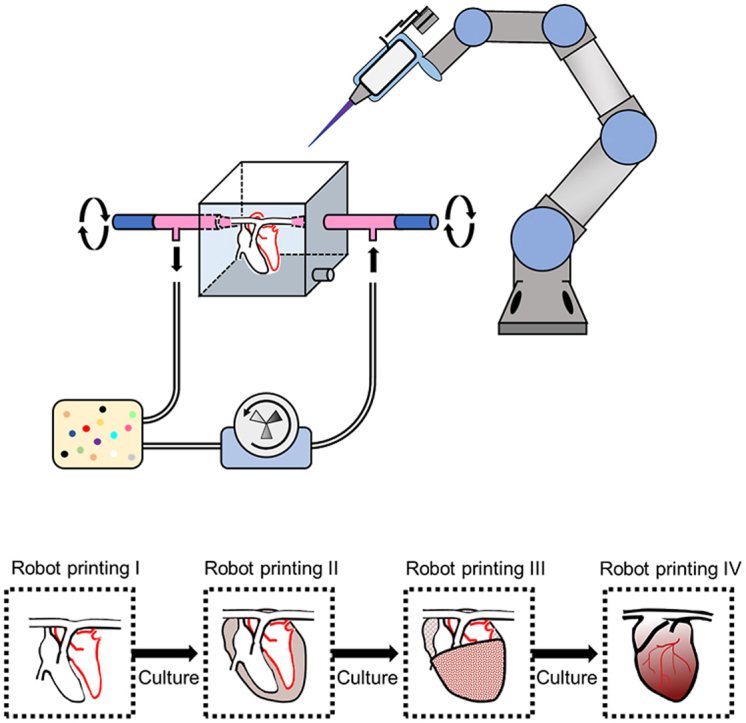

## 1 Introduction

Three-dimensional (3D) bioprinting is a promising approach to fabricate complex human tissues and organs, which are largely needed for regenerative medicine to treat organ-damage-related injury or diseases [1,2]. However, the current 3D bioprinting approaches still face difficulties in integrating printed cells with blood vessel networks to generate long-lived functional organs. The *in vivo* organ formation process requires blood supply for cells from the beginning of organogenesis [3,4]. Therefore, to obtain organs with physiological sizes and functions, the bioprinted cells must be interlaced and connected with blood vessel network to maintain long-term survival [5,6]. Yet the route to achieve such a goal still remains obscure. The commonly used 3D bioprinters are usually modified from layer-printing-based Cartesian 3D printers, which stack cells on simple-shaped flat planes with the assistance of polymerized biomaterials [1,2,6,7]. Such approach is unable to provide nutrient supplies to cells via blood vessel networks during the bioprinting process [5]. Furthermore, the addition of artificial biomaterials, which function as the “glue” to stick cells together, will inhibit the formation of functional cell-cell contact and angiogenesis among the already printed cells [8]. These limitations have become the major impediments for producing human-scale live organs via bioprinting, especially for printed cells/organs to achieve long-term survival and genuine functions [[6], [7], [8], [9]].

To solve these problems, we utilized a robotic arm with six degree-of-freedom (6-DOF) to build a new 3D bioprinting system. The robotic arm is composed of six 360° rotating joints, which has a greater flexibility than the planar layer-based 3D printers and is capable to place cells onto complex-shaped vascular scaffolds from all direction. Our robot-based 3D bioprinting system almost brings no harm to cells. In combination with a pre-constructed vascular scaffold, a self-designed bioreactor and a repeated print-and-culture approach, we were able to produce artificial blood vessels with *de novo* formed vascular branches and capillaries. We also used the bioprinting system to generate vascularized and contractable cardiac tissues, which maintained alive and beating for over 6 months. Combined application of two robotic arms enabled fast and accurate deposition of multiple cell types to from cell mixtures with designed patterns.

## 2 Materials and methods

### 2.1 Assembly and setup of the bioprinter

The bioprinter consists of two major parts: a 6-DOF robotic arm and a Multipette-based cell ejector. The desktop 6-DOF robotic arm used in this study (UR3) has a repeatability of ±0.1 mm and was bought from the Universal Robots company. In order to extend the reachability of the robotic arm, we mounted its base onto a lab-made stainless-steel pillar according to a previously published optimization method [10], then assembled the robotic arm on a stable working table, which provided a plane working surface and also minimized vibrations associated with the robot movement. We chose a single-channel Multipette (Eppendorf #M4) as the bioprinter for cell extrusion. The Multipette, together with a self-made stepper motor or an electromagnetic thruster, was mounted to the end-effector of the robotic arm by a 3D printed resin fixture (Fig. S2). The stepper motor or the electromagnetic thruster controlled the extrusion of bioink through the Multipette, and the action of the stepper motor or the electromagnetic thruster was operated by an Arduino board via in-house developed C++ scripts.

### 2.2 Assembly and setup of the bioreactor

The self-designed bioreactor consists of two major parts, a tank within which the cells were printed and the bioprinted or post-printed cells/tissues were cultured, and a nutrient circulator connecting the vascular scaffold and external medium with latex tubes. The self-manufactured trapezoid tank was made of titanium. To build a circulation loop for the bioreactor, we installed a leakproof bearing (Φ = 8 mm) holding a short rotatable hollow rod onto each of the short side wall of the tank. The inner end of each rod within the tank was used to insert into the terminal lumen of the tubular scaffold, and the rod ends outside of the tank each had a side-opening to connect to a latex tube. By connecting both latex tubes to the same peristaltic pump (QiTe #BT1-100 V-LCD) with cell nutrient medium, the tubular scaffold, the rods, and the latex tubes could form a closed circulation to provide nutrients for cells attached on the tubular scaffold. To enable rotation of the tubular scaffold for multi-directional bioprinting, the rod ends outside of the tank were each connected to a rotation motor. The motors were supported by 3D-printed resin fixtures (UnionTech Lite 600) and were connected to the same Arduino board controlling the stepper motor mentioned above. The tank was fixed on top of a heating block (Inheco TEC) to ensure stable temperature (usually 37 °C) during the long-term culture process. The assembled bioreactor was covered by a removable acrylic lid and installed onto a stainless-steel board, and the steel board was then mounted to the working table at defined coordinates. The entire bioprinting system was set up in an ISO Class 5 cleanroom (ISO 14644–1) equipped with two independent airways to keep the hygiene environment and to maintain consistent CO_2_ concentration (5%) in the air.

### 2.3 Computational control of the bioprinting system

The entire bioprinting system was controlled by in-house developed C++ scripts. To precisely control the movement of the robotic arms, we first designed the printing paths according to the shapes of different objects/scaffolds. The printing target locations on the scaffold were extracted and considered as a list of Cartesian coordinates, which were smoothened to generate the movement paths for the robotic arms. The movement trajectories and poses of the robotic arms toward each printing position were computed by inverse kinematics, collisions were avoided by methods reported in a previous publication [11]. After reaching each target position, the Arduino board would follow the command of the C++ scripts to initiate the motion of the stepper motor to print cells.

### 2.4 Fabrication of printing scaffolds

**Fig. 1:**
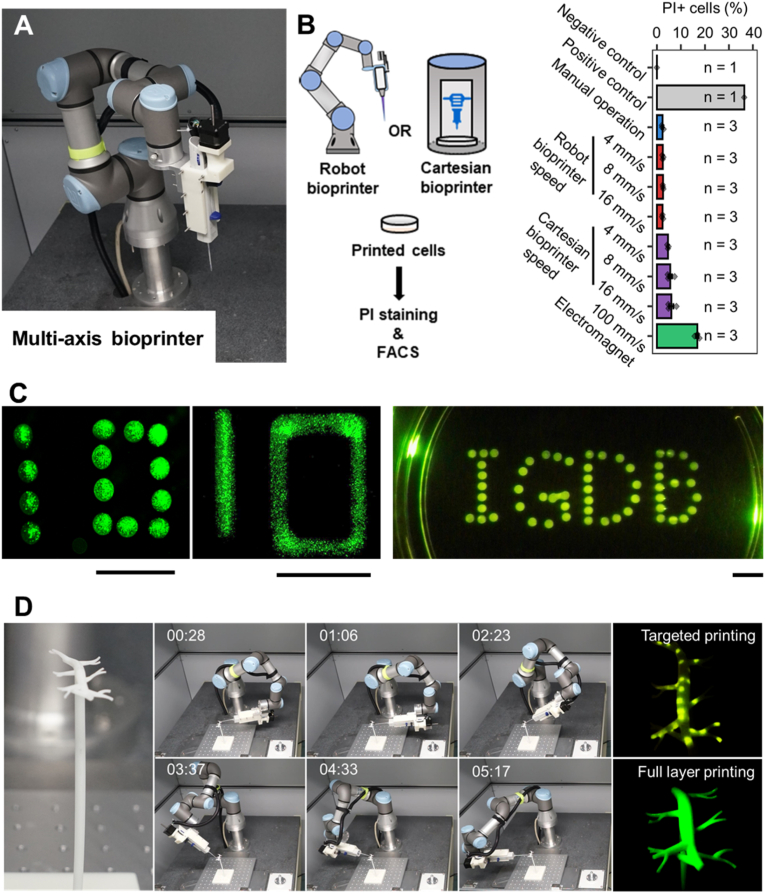
Six-DOF robot bioprinter brings no damage to cells and supports multi-dimensional cell printing. (**A**) Composition of the 6-DOF robot bioprinter. An Eppendorf Multipette equipped with a stepper motor is mounted onto the end-effector of a 6-DOF robot to assemble as a 3D bioprinter. (**B**) Mechanical damage assessment of hCMEC/d3 cells printed by a 6-DOF robot bioprinter, a Cartesian bioprinter or an electromagnet cell ejector with different printing speeds. Shown are the schematic display of the experimental workflow (left part) and the proportion of propidium iodide (PI) staining positive cells measured by FACS. Negative control, cells without PI staining; positive control, cells pre-treated with Camptothecin (CPT, 1 μM for 24 h) to induce massive cell damage. n represents numbers of independent trials. Error bars, standard error of the mean. See also Fig. S4 for representative FACS charts. (**C**) Printed eGFP-labeled hCMEC/d3 cells resembling discrete “10”, continuous “10” and IGDB (from left to right). Scale bars, 1 cm. (**D**) Process (middle) and final outcome (right) of printing hCMEC/d3-eGFP cells onto a complex vascular scaffold (left). Numbers at the upper-left corner of the middle panels are the timestamps during the photo recording process.

The resin scaffolds of 3D blood vessel models (in Fig. 1D), 3D human heart model (in Fig. S5C) and complex-shaped coronary scaffolds (in Fig. 6) were printed by a stereolithographic 3D printer (UnionTech Lite 600). The tubular poly l-lactic acid (PLLA) scaffolds used in cell bioprinting experiments were fabricated by electrospinning method (Ucalery). In brief, PLLA (average molecular weight = 1.7 × 10^6^ g/mol) was dissolved in a binary solvent system composed of methylene chloride and *N*, *N*-dimethylformamide (v/v = 50/50) to obtain a polymer concentration of 10% w/v. The PLLA solution was placed in a plastic syringe and pumped out to form a spray with a constant feeding rate of 1 ml/h under the voltage of 15 kV. A self-made rod-shaped copper collector (Φ = 2 mm) covered with aluminum foil was placed 10 cm away from the syringe to collect the electrospun PLLA fibers. After collection, the PLLA scaffolds were air dried and carefully separated from the collector.

### 2.5 Cells and reagents

Experiments involving human embryonic stem cells (H9) were designed in compliance with the ISSCR 2016 guidelines and were reviewed by the IGDB Institutional Review Board. All cells were cultured at 37 °C with 5% CO_2_ and were routinely tested to avoid mycoplasma contamination by PCR (GeneCopoeia #MP001). HeLa cells (ATCC #CCL-2) and MOVAS cells (ATCC #CRL-2797) were routinely cultured in the DMEM medium (Gibco #11965084) supplemented with 10% fetal bovine serum (FBS, Biological Industries #04-001-1A) and 1 × antibiotic-antimycotic solution (Gibco #15240062). hCMEC/d3 endothelial cells (Merck #SCC066) were routinely cultured in the RPMI-1640 medium supplemented with 10% FBS, 1 × antibiotic-antimycotic solution, 1 × NEAA (Gibco #11140076) and 1 × GlutaMax (Gibco #35050061). These cells were passaged every 3 days using 0.25% Trypsin (Gibco #25200056).

H9 hESCs (WiCell #WA09) were maintained following the WiCell guidelines. In brief, H9 cells were routinely cultured in TeSR-E8 complete medium (StemCell #05990) on 1% Matrigel (Corning #354277) coated 6-well plates and were passaged with ReLeSR (StemCell #05872) every 4 days. H9 cells used in this study were below passage 50. Differentiation of cardiomyocytes from hESCs were performed following a Wnt pathway-based publication [12] with minor modifications. In brief, H9 colonies were digested by Accutase (Merck #SCR005) and plated on 1% Matrigel-coated 6-well plates at a density of 1 × 10^6^ cell/ml in TeSR-E8 complete medium + 2 μM Y-27632 (StemCell #72304). The medium was replaced with TeSR-E8 24 h after plating and daily refreshed afterwards. The growth of H9 cells was monitored under a microscope till the colonies reached 90% confluency, which was noted as day 0 for cardiomyocyte differentiation. Cells were cultured with RPMI-1640 medium (Gibco #11875085) supplemented with 2% B27 minus insulin (Gibco #A1895601) and 8 μM CHIR-99021 (Tocris #4423/10) from day 0 to day 2, RPMI-1640 supplemented with 2% B27 minus insulin and 2 μM Wnt-C59 (Selleck #S7037) from day 2 to day 4, and RPMI-1640 supplemented with 2% B27 (Gibco #17504044) from day 4 to day 12 (refreshed every 2 days). Contracting cardiomyocytes were observed on day 10 and were purified on day 12 by switching the medium to glucose-free RPMI-1640 (Gibco #11879020) supplemented with 2% B27 and 5 mM lactate (Sigma #L4263) [13]. The lactate medium was daily refreshed to remove dead non-cardiomyocyte cells and was switched back to RPMI-1640 (Gibco #11875085) + 2% B27 medium on day 15.

### 2.6 Cell survival evaluation after bioprinting

As high cell ejection speeds may cause bioink to splash and damage the printed cells, we screened for an optimal bioink ejection speed of our robot bioprinter and compared it with an extrusion-based Cartesian bioprinter. We prepared endothelial cell bioink by suspending normally cultured hCMEC/d3 cells in culture medium consisting of RMPI-1640 (Gibco #11875085) + 10% FBS with a concentration of 5 × 10^6^ living cells/ml, and printed the bioink using either our stepper motor-driven robot bioprinter (nozzle diameter = 200 μm) or a Cartesian bioprinter (nozzle diameter = 200 μm) with extrusion speeds of 4 mm/s, 8 mm/s and 16 mm/s. To estimate cell damages caused by manual operation or ultra-high bioprinting speed, bioinks were also manually operated using an Eppendorf pipette or printed by a high-speed electromagnetic thruster (printing speed = 100 mm/s, nozzle diameter = 200 μm). All printing experiments were repeated 3 times. In each experiment, the printed cells were immediately stained with 3 μM propidium iodide (PI, Solarbio #P8080) for 10 min at 37 °C, then washed with 1 × PBS twice and subjected to FACSAriaII analysis for PI^+^ cells. To generate positive controls for cell damage measurement, a dish of hCMEC/d3 cells were treated with 1 μM Topoisomerase inhibitor Camptothecin (CPT) for 24 h to induce cell death, then stained by PI.

To measure the viability of printed human cardiomyocytes (Figs. S6B-D), we carefully collected H9-differentiated cardiomyocytes on post-differentiation day 18 using Cardiomyocyte Dissociation Medium (StemCell #05026) and prepared bioink by resuspending the cells in Cardiomyocyte Support Medium (StemCell #05027) to reach a concentration of 5 × 10^6^ live cells/ml. The bioink was then either gently hand-seeded or robot-printed (printing speed = 8 mm/s) into the culture medium in 1% Matrigel-coated plates. Cardiomyocytes were cultured at 37 °C with 5% CO_2_ for 24 h, stained by Trypan Blue (Thermo #15250061) and counted by Countess II FL (Thermo).

### 2.7 Immunohistochemical analysis of printed cells/tissues

To prepare cell samples for immunohistochemical analysis, bioinks (cardiomyocytes, hCMEC/d3 or HeLa cells, 5 × 10^6^ cells/ml) were printed or manually plated onto poly-d-lysine-coated coverslips and cultured for 24–48 h. Coverslips were fixed with 4% PFA for 10 min at room temperature (RT), washed with 1 × PBS for 3 times, then permeabilized and blocked with 0.1% Triton X-100 dissolved in 2% BSA (1 × PBS) for 10 min at RT.

To prepare tissue sections for immunohistochemical analysis, tubular scaffolds were fixed in 4% PFA overnight with gentle rocking at 4 °C, washed by 1 × PBS for 3 times (10 min each), then dehydrated by 30% sucrose overnight at 4 °C. Dehydrated printed tissues were embedded in TFM (General Data #TFM-5), frozen at −20 °C and sectioned using a Leica cryostat microtome (15–20 μm per section, Leica #CM3050S). Glass slides carrying tissue sections were washed with 1 × PBS to dissolve the TFM, permeabilized and blocked with 0.1% Triton X-100 in 2% BSA for 20 min at RT.

Permeabilized coverslips and glass slides were incubated with the primary antibodies (diluted in 0.1% Triton X-100 + 2% BSA) overnight at 4 °C, washed with 1 × PBS for 3 times, then incubated with the secondary antibodies for 1 h at RT, counterstained with 0.5 μM DAPI (Abcam #ab228549) for 15 min at RT, and washed with 1 × PBS for 3 times. Coverslips and slides were mounted using Prolong Gold Antifade Reagent (Thermo #P36930) and imaged using a Leica TCS SP8 confocal microscope. Z-stacks were generated using Leica LAS X software (v. 2.0.1.14392). Images were analyzed using ImageJ software (v. 1.51k). Antibodies used in this study are listed in the Supplementary Materials.

### 2.8 Cell cycle analysis

To analyze whether our bioprinter would bring any changes to cell cycle, HeLa and hCMEC/d3 bioinks (5 × 10^6^ cells/ml in DMEM + 10% FBS or RPMI-1640 + 10% FBS respectively) were either manually seeded or printed into Petri dish and cultured for 48 h at 37 °C with 5% CO_2_. H9 hESCs cultured on Matrigel-coated 6-well plates were continuously passaged using ReLeSR (4 days per passage) for 5 times by either manual operation or robot printing. EdU (10 μM, Thermo #C10337) was added to the cell culture medium (DMEM + 10% FBS for HeLa cells, RPMI-1640 + 10% for hCMEC/d3 cells, and TeSR-E8 for H9 hESCs) for 1 h to label nascently synthesized DNA. The cells were then washed by 1 × PBS for 3 times, digested by either 0.25% Trypsin (HeLa and hCMEC/d3 cells) or Accutase (H9 cells) for 3 min at 37 °C, and washed by 1 × PBS once. After that, cells were collected and fixed by 4% PFA for 10 min, permeabilized by 0.1% Triton X-100 for 10 min, and washed by 1 × PBS for 3 times. Cells were then incubated with the staining solution from Click-iT EdU Alexa 488 kit (Thermo #C10337) for 30 min, counterstained by 0.1% Hoechst 33342 (Thermo #998468) for 10 min, then washed by 1 × PBS for 3 times, and measured by FACSAriaII. Experiments after fixation were performed under RT.

### 2.9 Printing on different ECMs under oil bath

To evaluate the attachment ability of cell culture medium on different ECMs under the oil bath, we prepared different ECM-coated surfaces as follows: nonadherent plastic flasks were filled with one of the following materials, namely poly-d-lysine (100 μg/ml, Solarbio #P2100), fibronectin (50 μg/ml, Merck #FC010), gelatin (50 μg/ml, ADI #GELB11), collagen I (50 μg/ml, Thermo #A1048301), and Matrigel (1%) diluted in ice cold RPMI-1640 medium without phenol red (Thermo #11835030), and incubated at RT for 24 h. Then the coating solutions were discarded, and the flasks were air dried at RT for 24 h and filled with embryo grade mineral oil (OVOIL #10029). Cell culture medium (RPMI-1640 + 10% FBS without cells, 1 μl per drop) was printed onto the inner vertical surface of oil-filled flasks, which were then kept still at RT for 24 h. The attachment status of RPMI-1640 medium drops to different ECM surfaces was recorded right after printing and after 24-h incubation at RT using a digital camera equipped with a micro-lens (Sony # SEL50M28).

### 2.10 Screening for optimal cell concentration in bioink

hCMEC/d3 cells were suspended in RPMI-1640 medium consisting of 10% FBS at various concentrations and then printed onto Petri dishes under oil bath: 1 μl bioink consisting of 100, 500, 1000 or 5000 cells were each incubated for different time intervals (10 min, 30 min, 60 min, 4 h or 16 h) at 37 °C with 5% CO_2_. After incubation, cells were washed by 1 × PBS for 3 times to remove any nonattached or weakly attached cells. Cells remained on the plates were stained with the Live-Dead Cell Staining Solution (Solarbio #CA1630), which marked live cells with activated Calcein-AM and dead cells with PI. Stained cells were imaged using a fluorescence microscope (Leica DMI 3000B).

### 2.11 Preparation of tubular scaffold and setup of bioprinting environment

Before performing the bioprinting experiments, we first sterilized the tubular scaffold and the bioreactor. Tubular PLLA scaffold was connected to the needle pins at both ends, fastened by suture, then submerged in 75% ethanol for 1 min and washed by 1 × PBS + 3 × antibiotic-antimycotic solution for 3 times for sterilization. The sterilized PLLA scaffold was precooled in cold RPMI-1640 basal medium, then coated with Matrigel by incubating in 1% Matrigel solution (diluted in RPMI-1640 basal medium) at 37 °C overnight. To sterilize the bioreactor, tank and circulation loop were first perfused with 75% ethanol for 1 h, then perfused with 1 × PBS + 3 × antibiotic-antimycotic solution for 1.5 h (the entire perfusion medium was refreshed every 0.5 h). We next assembled the Matrigel-coated tubular scaffold onto the rotation rods inside the bioreactor by screwing the needle pin into the rod opening, then filled the tank and perfused the circulation loop with buffer medium (basal RPMI-1640 medium + 3 × antibiotic-antimycotic solution) overnight. The bioreactor at this stage was ready for cell perfusion and cell printing experiments.

### 2.12 General process of bioink preparation for bioprinting on the tubular scaffolds

hCMEC/d3, H9 hESCs and differentiated cardiomyocytes were cultured as mentioned above. To prepare bioink consisting of endothelial cells, hCMEC/d3 cells reaching 80% confluency were digested by 0.25% Trypsin for 3 min at 37 °C, followed by adding equal volume of RPMI-1640 + 10% FBS medium to terminate the digestion, then collected and centrifuged at 1000 rpm for 3 min. The collected hCMEC/d3 cells were washed with 1 × PBS once, counted by Countess II and resuspended in appropriate medium at defined cell concentration (see Supplementary Materials for more details).

To prepare bioink consisting of hESCs, H9 colonies cultured on Matrigel-coated 6-well plates with 80–90% confluency were dissociated by Accutase digestion for 5 min at 37 °C, the digestion was terminated by adding equal volume of TeSR-E8 medium to the plates. Cells were collected and centrifuged at 800 rpm for 3 min, and then washed by 1 × DMEM/F12 (Cellgro #10-092-CV) once, counted by Countess II and resuspended in appropriate medium at defined cell concentration (see Supplementary Materials for more details).

To prepare bioink consisting of cardiomyocytes, H9 hESC-differentiated cardiomyocytes were harvested from the 18th to 20th day of differentiation by using the Cardiomyocyte Dissociation Medium following the manufacturer's instruction. Collected cardiomyocytes were centrifuged at 800 rpm for 5 min, washed by basal RPMI-1640 medium once, counted by Countess II and resuspended in appropriate medium at defined cell concentration (specified in Supplementary Materials).

### 2.13 General process of bioprinting on the tubular scaffold

In this study, all bioinks were printed at RT and post-printing cultured at 37 °C. To print bioinks onto the tubular scaffolds mounted in the tank, we first filled the tank with embryo grade mineral oil (OVOIL #10029) to create a hydrophobic printing environment. During the printing process, the lumen of the tubular scaffold was filled with RPMI-1640 medium supplemented with 10% FBS, 1 × antibiotic-antimycotic solution and 10 mM HEPES. We then calibrated the robot position by adjusting the linear distance between the printing tip and the scaffold to 1 mm and avoiding collisions at the meantime. After calibration, we returned the robot to its standby position, loaded bioink into the Multipette, and initialized the printing program. To print on cylindrical tubular scaffolds, the entire surface of the scaffold was divided into 3 equal parts along the longitudinal axis, each part was designed to be covered by 4 printing paths. At each ejection of the Multipette, 1 μl bioink was printed onto the scaffold with the assistance of oil bath. The robot was programed to cover each part of the scaffold (with 4 paths) in a zigzag manner at a moving speed of 1 mm/s. At the transition position between two joining paths, the robot was programed to lift away from the scaffold surface for 2 cm and then shift its orientation to the next path to avoid collision. Upon finishing printing the upper 1/3 area (first part), the system would pause for 20 min to wait for the cells to form stable attachment to the scaffold, then invoked the rotation of the scaffold to slowly switch the second 1/3 part upward in a speed of 0.268° per second, after which the robot resumed its initial printing position and printed the next 1/3 part. After finishing printing the entire scaffold, the robot would return to its standby position. See Supplementary Materials for detailed method description on bioprinting of artificial blood vessel, vascularized cardiac tissue and human embryonic stem cells.

### 2.14 General procedures for post-printing cell culture

Upon finishing the bioprinting and cell incubation, we replaced the oil bath in the bioreactor with appropriate culture medium according to the type of printed cells (specified in Supplementary Materials) and cultured the cell-covered scaffolds at 37 °C with 5% CO_2_ for 2 h. Then we connected the perfusion tubes with the bioreactor and started perfusion with corresponding culture medium (specified in Supplementary Materials) at a speed of 1 ml/min, and continued to culture the printed tissue at 37 °C with 5% CO_2_ for time intervals as needed. For tasks requiring repeated bioprinting, the printing process would be reperformed at 24 h after the previous printing step. For long-term culture of the cell-covered scaffolds, the rotation function of the rods would be turned on at the 1st day after finishing all bioprinting processes to create a condition mimicking 3D cell culture. The rods were programmed to repeatedly rotate clockwise for 240° then anticlockwise for 240° at a speed of 0.268° per second. For long-term culture, the culture medium in the bioreactor and the circulation loop was refreshed every 7 days.

### 2.15 Collaborative printing by two robot bioprinters

As described in section 2.1, we assembled two UR3 robot-based bioprinters on the diagonal margins of a stable working table, and simultaneously controlled the motion of both robots through Arduino board with in-house developed C++ scripts. The two robots were individually loaded with different bioinks: one was loaded with hCMEC/d3 cells (5 × 10^6^ cells/ml in RPMI-1640 + 10% FBS + 1% Matrigel) stained by 1% Fast Green (Sigma #F7252), the other was loaded with H9-differentiated cardiomyocytes (5 × 10^6^ cells/ml in RPMI-1640 + 10% FBS + 1% Matrigel) stained by 1% Neutral Red (Macklin #N814728). We programmed the two robots to collaboratively print cells onto a complex-shaped coronary scaffold submerged under oil bath. The moving speed of both robots was set as 1 mm/s.

## 3 Results

### 3.1 Design of a 6-DOF robot-based bioprinting strategy to support multidirectional cell printing

In order to tackle the long-term survival issue of cells in bioprinted organs, we designed a 6-DOF robot-based print-and-culture system to enable nutrient supply to cells during and after the bioprinting process. The system is composed of a 6-DOF robotic arm with a cell ejector as the bioprinter, a synthesized vascular scaffold to serve as the cell attachment frame and nutrient delivery pipe, an oil-based hydrophobic printing environment to facilitate the attachment of printed cells to the vascular scaffold, as well as a self-designed bioreactor equipped with heating and circulation devices to nurture cells during and after the bioprinting process (Fig. S1). Such a system enables cell printing on complex-shaped vascular scaffolds, as well as prolonged intervals between bioprinting rounds to assist the formation of capillaries and functional cell-cell connections among printed cells. By repeating this print-and-culture process, large-scale bioprinted artificial organs could be generated (Fig. S1).

To convert the 6-DOF robot into a bioprinter, we first designed and installed a cell ejector onto the end-effector of the robotic arm (Fig. 1A and Figs. S2A–C). The cell ejector is a stepper motor operated Eppendorf Multipette with the corresponding pipette tip as the nozzle (Fig. S2D), which continuously print cells after one bioink fill (Fig. S2E and Video S1). The cell ejector was connected to the robotic arm by a customized fixture (Figs. S2B and C), the motions of the stepper motor and the robotic arm were both operated by an Arduino board via in-house developed C++ scripts (Fig. S3). To measure cell damages associated with the printing process, we suspended human endothelial cells (hCMEC/d3 cells, hereafter termed as hECs) in culture medium to sever as the bioink, then printed the bioink with various printing speeds (4, 8 and 16 mm/s), and measured cell damage by propidium iodide (PI) staining (Fig. 1B). For comparisons, we also printed hECs with an extrusion-based Cartesian bioprinter using the same printing speeds (4, 8 and 16 mm/s) and a high-speed electromagnet-driven cell ejector (100 mm/s, Video S2). PI positive cells were only about 2% for our robot bioprinter at all tested printing speeds, which were similar to the cell damage ratio associated with manual operation (Fig. 1B and Fig. S4). Yet cell damage caused by the Cartesian bioprinter was ∼4% at the printing speed of 4 mm/s, and increased slightly with the increment of printing speed (Fig. 1B and Fig. S4). The high electromagnet-driven cell ejection (100 mm/s, Video S2) caused severe cell damages, demonstrating the advantage of our designed stepper motor in cell printing (Fig. 1B and Fig. S4). Basing on these results, we chose 8 mm/s as the printing speed of our robot bioprinter.

Supplementary data related to this article can be found at https://doi.org/10.1016/j.bioactmat.2022.02.009.

The following are the supplementary data related to this article:

Multimedia component 2 Multimedia component 2

Multimedia component 3 Multimedia component 3

The 6-DOF robot can move and twist to reach any angle within a 3D space (Video S3), thus is capable to print cells along all directions. We first tested the flexibility and controllability of our robot bioprinter by drawing a coronary blood vessel network on a resin human heart model (Figs. S5A–C and Video S4), then printed eGFP labeled hCMEC/d3 cells (hEC-eGFP) via tracks representing either dispersed or continuous “10”, as well as the letters “IGDB” (abbreviation for Institute of Genetics and Developmental Biology) on horizontally placed Petri dishes (Fig. 1C). Furthermore, we programmed the bioprinter to print hEC-eGFP cells on a complex-shaped 3D vascular scaffold at defined spots or to form a full coverage through various paths (Fig. 1D and Fig. S5D), demonstrating the capability of our robot bioprinter to carry out complicated tasks.

Supplementary data related to this article can be found at https://doi.org/10.1016/j.bioactmat.2022.02.009.

The following are the supplementary data related to this article:

Multimedia component 4 Multimedia component 4

Multimedia component 5 Multimedia component 5

### 3.2 The robot bioprinter fully preserves cell viability and activity

**Fig. 2:**
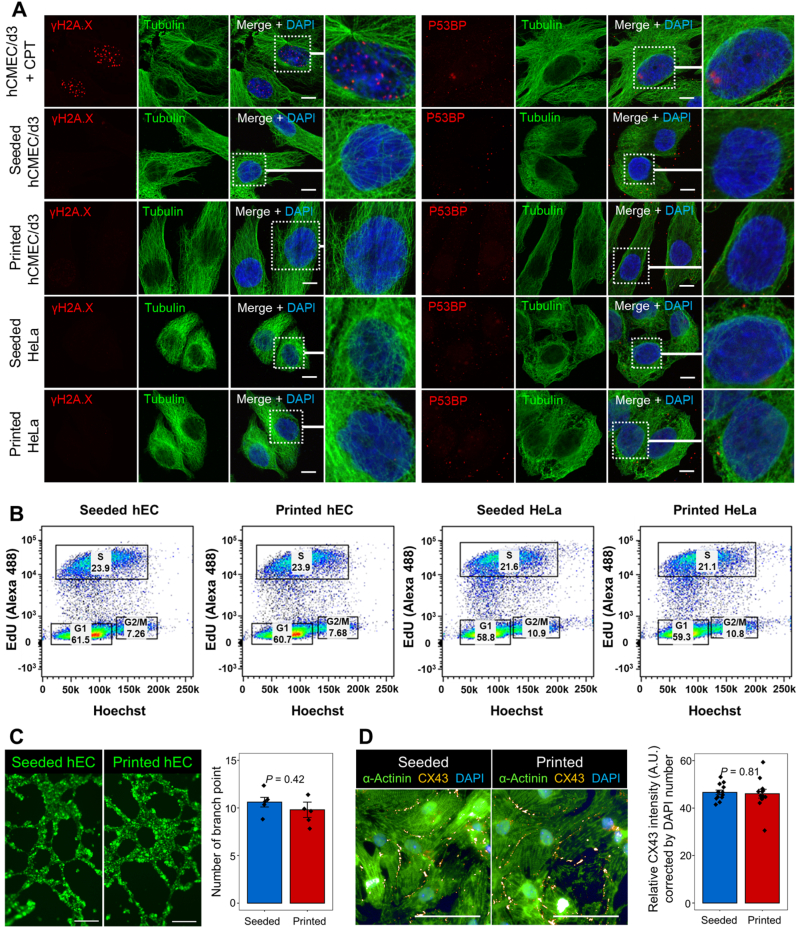
Six-DOF robot-based bioprinting preserves genome integrity, proliferation capacity and biological activity of printed cells. (**A**) Immunohistological analysis of DNA damages of manually seeded and robot printed hCMEC/d3 cells or HeLa cells. Damaged foci are marked by γH2A.X (left) and P53BP (right). CPT treated hCMEC/d3 cells are used as DNA damage controls. (**B**) Robot printed cells and manually seeded cells have similar cell cycle distributions. **(C)** Tubular formation comparison between manually seeded and robot printed hCMEC/d3-eGFP cells. (**D**) Gap junction density comparison between manually seeded and robot printed cardiomyocytes. n = 5 replicates in (**C**) and 12 replicates in (**D**). Error bars, standard error of the mean, *P*-values are calculated by Student's *t*-test. Scale bars, 10 μm in (**A**), 250 μm in (**C**) and 50 μm in (**D**).

To examine whether the bioprinting operation would bring long-term side effects on cell proliferation and activity, we cultured robot printed or manually seeded HeLa cells and hECs for 48 h, then examined their genome integrity. No difference on DNA damage or cell cycle distribution was detected between the robot printed and manually seeded cells (Fig. 2A and B, and Fig. S6A). In addition, both manually seeded and robot printed hECs formed well-developed vascular network with similar numbers of branch points after 24-h culture on Matrigel, demonstrating the well-preserved angiogenesis ability of endothelial cells after printing (Fig. 2C). As cardiomyocytes are highly vulnerable to mechanical stress, we next printed human embryonic stem cell (hESC)-differentiated cardiomyocytes (hCMs), and found no additional damage among printed cells as compared with the manually seeded ones after 24 h (Figs. S6B-D). Similar cardiac gap junction [14] densities were detected among the printed and manually seeded hCMs (Fig. 2D), and both groups of cells resumed contraction after 48 h, further proved the good performance of our robot bioprinter in preserving the viability and functions of printed cells.

### 3.3 The robot bioprinter prints cells in multiple directions with the assistance of oil bath

**Fig. 3:**
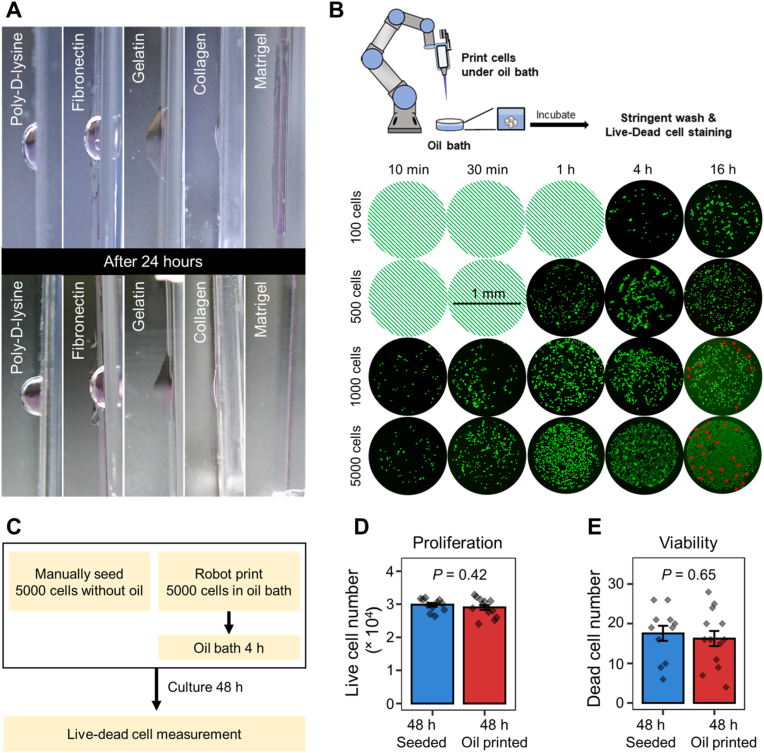
Oil bath maintains the round shape of aqueous droplets after printing. (**A**) The attachment of 1 μl aqueous droplet (culture medium, RPMI-1640 + 10% FBS) onto the extracellular matrix protein coated vertical plastic surface with the assistance of oil bath. (**B**) Screening for optimal cell concentrations of the bioink. Upper panel, experimental setup diagram. Lower panel, attachment and survival examination of hCMEC/d3 cells (1 μl) with various concentrations after printing and oil incubation. Green dots, fluorescence of Calcein-AM for living cells; red stars, indicators for propidium iodide positive dead cells (see also Fig. S7D for magnification of red star-marked dead cells). Light green-colored circles represent plates without cells. (**C**) Experimental setup diagram for evaluating the effects of oil incubation. (**D**) Cell proliferation comparison of samples treated by (**C**). (**E**) Dead cell comparison of samples treated by (**C**). Error bars, standard error of the mean. *P*-values are calculated by Student's *t*-test.

Unlike the conventional Cartesian bioprinters which usually print cells layer-by-layer in a bottom-up manner [7], our 6-DOF robot-based bioprinter is capable to print cells toward any directions. To avoid cell damages brought by the commonly used self-consolidation materials [2], we developed a hydrophobic cell printing environment to print aqueous bioink under an oil bath along any direction and utilized the hydrophobic-hydrophilic force to keep printed cells attached to the complex-shaped scaffolds. The scaffolds were pre-coated with extracellular matrix proteins to facilitate cell attachment in oily environment without the assistance of other fixation biomaterials, and the oil bath was switched to cell culture medium after full attachment of the cells (Fig. S7A). To screen for suitable ECM proteins, we first coated the inner surface of plastic flasks with poly-d-lysine (PDL), fibronectin, gelatin, collagen, or Matrigel, then filled the flasks with mineral oil to create a hydrophobic environment (Fig. S7B). After printing 1 μl cell culture medium onto the vertical inner surface of the flasks, all coating proteins supported the stable attachment of the bioink to the coated vertical surfaces (Fig. 3A and Fig. S7C). In particular, the printed culture medium stretched to form a monolayer on Matrigel coated surface, therefore could better facilitate the attachment of newly printed cells with the previously printed ones (Fig. 3A and Fig. S7C). We thus chose Matrigel as the coating material for the bioprinting scaffolds.

As our bioprinting strategy utilize a repeated print-and-culture method to assist vascularization among printed cells, we then screened for the optimal cell concentration of bioink and the suitable post-printing incubation duration under the hydrophobic bioprinting condition. We printed 1 μl bioinks containing either 100, 500, 1000 or 5000 hECs under oil bath and incubated the printed cells in the oil bath for 10 min, 30 min, 1 h, 4 h, or 16 h. Under these conditions, nutrients for cells were only supplied by the culture medium in the 1 μl bioink. After incubation, cells were washed with PBS and measured for viability by live-dead cell staining in RPMI-1640 medium (Fig. 3B). The results showed that the longer the incubation time, the more cells were attached to the supporting surface (Fig. 3B). For all tested incubation durations, 4 h or less preserved cell viability well, and dead cells were only detected in the 16 h incubation samples (Fig. 3B, Fig. S7D). We further cultured the 4 h oil-incubated cells in normal hEC culture medium for another 48 h, and found no difference between these cells and the manually seeded cells without oil treatment in terms of both cell proliferation and survival rates (Figs. 3C–E). We further found that adding 1% Matrigel into the bioink could improve cell attachment and generate firmly attached cell monolayer on the supporting surface after oil incubation for only 30 min (Fig. S7E). Basing on these optimizations, we chose ∼5000 cells/μl plus 1% Matrigel as our bioink setting, and oil incubation for 30 min as the cell attachment condition.

### 3.4 Design of a bioreactor to support efficient multidirectional printing and cell culture

**Fig. 4:**
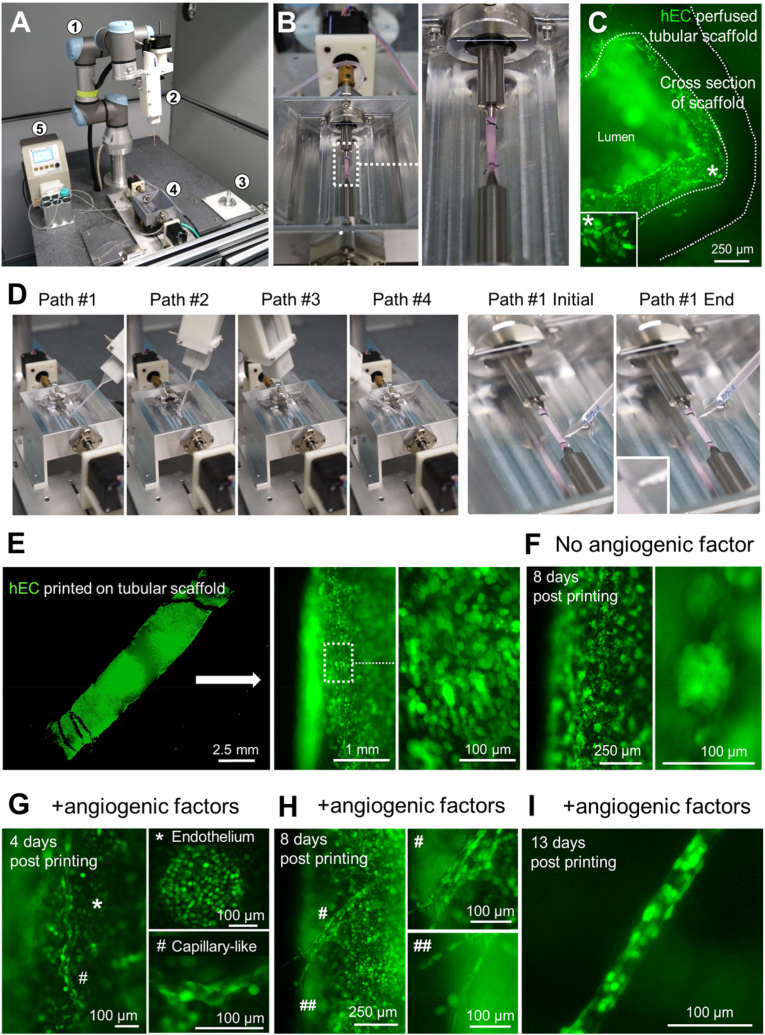
Printed artificial blood vessel is capable of angiogenesis and vasculogenesis. (**A**) Overview of the entire bioprinting platform (① 6-DOF robot; ② Multipette-based cell ejector; ③ calibration pin; ④ bioreactor; ⑤ circulation pump). (**B**) Setup of the perfusion test. The bioreactor tank is filled with mineral oil, the tubular scaffold (originally white color, see Figs. S10D and E) is perfused with RPMI-1640 medium (phenol red) + 10% FBS and hCMEC/d3-eGFP cells. (**C**) Cross-section of the perfused tubular scaffold in (**B**). See also Fig. S10F for full view. (**D**) Recordings of the cell ejector positions at each printing path of the first print round for artificial blood vessel bioprinting. Inset in the last panel is the snapshot of a bioink drop (hCMEC/d3-eGFP cells) at the nozzle tip before attaching to the scaffold. (**E**) Fluorescent overview of the printed artificial blood vessel at 24 h after 2 cycles of print-and-culture process (left) and its zoom-in view (middle and right). (**F**) Artificial blood vessel cultured in the bioreactor for post-printing 8 days shows overgrowth of hCMEC/d3-eGFP cells (left) and its zoom-in view (right). (**G** to **I**), Post-printed artificial blood vessel cultured in the bioreactor with perfusion of angiogenic factors (combination of VEGF, FGF, EGF and IGF, see Supplementary Materials and Methods) for 4 days (**G**), 8 days (**H**), and 13 days (**I**). The newly formed endothelium and capillary-like structures are marked by asterisks and pound signs in **G** and **H**, respectively. Typical capillary-like structures can be observed in **I**. See also Fig. S11D for two more examples.

In order to obtain functional *in vitro* manufactured organs, the printed cells should be cultured during bioprinting intervals to allow the formation of cell-cell contact and blood vascular networks. To facilitate repeated cell print-and-culture, we designed a multifunctional bioreactor, which is composed of a thermostatic square-shaped tank equipped with a pair of rotatable short hallow rods (Figs. 4A and B, and Fig. S8A). The rods were used to connect synthesized vascular scaffolds (a piece of synthesized vessel in this experiment) with an external pump, which enables blood or cell culture medium to consistently flow through the vascular scaffolds to provide nutrient supports to printed cells (Fig. S8B). To ensure even distribution of printed cells on all surface of the scaffold, we applied a print-and-rotate strategy in each print-and-culture cycle (Figs. S9A and B). Taking the tubular scaffold as an example, its entire cylindrical surface was divided into 3 equal parts along the longitudinal axis. After printing cells on each part with 4 adjacent and parallel printing paths (30° interval), the system remained static for certain period (e.g., 30 min) to enable the printed cells to attach to the scaffold, then gradually rotated the rods to bring the next 1/3 surface upward and started the next round of bioprinting (Fig. S9). By repeating such procedure, cells were printed onto the entire surface of the vessel (Fig. S9). If equipped with a larger tank and more complicated vascular scaffold, more complex artificial organs with bigger sizes could be generated.

### 3.5 The print-and-culture strategy enables vasculogenesis and angiogenesis of artificial blood vessels

After installation of the above-described robot bioprinting system (Fig. 4A), we designed a general workflow (Fig. S10A) to fabricate artificial blood vessels. The poly l-lactic acid (PLLA) tubular scaffolds were first sterilized and coated with Matrigel, connected to the rotating rods inside the bioreactor, we then filled the bioreactor with mineral oil (Figs. S10B–E). To create an endothelial inner layer for the blood vessel, we perfused hEC-eGFP cell suspensions at a concentration of 10^6^ cells/ml through the scaffold lumen for 24 h (Fig. 4B). Fluorescence detection showed that hEC-eGFP cells attached well to the inner wall of the scaffold (Fig. 4C and Fig. S10F). No fluorescence signal was detected in mineral oil or on the outer surface of the scaffold (Fig. 4C), proving that the PLLA scaffold, together with the bioreactor, formed an enclosed circulation, and the perfusion of hECs produced endothelium layers inside the tubular scaffold.

To evaluate the long-term survival and vascularization ability of endothelial cells after printing, we prepared endothelial bioink and printed them onto the outer surface of a Matrigel-coated vascular scaffold for 2 print-and-culture cycles (Fig. 4D, Figs. S10G, S11A and B, and Video S5), then cultured the printed vessels inside the bioreactor with gentle rotation. RPMI-1640 medium with or without angiogenic factors was perfused through the inner lumen of the vessel (Fig. S11C). To avoid interference of fluorescence signals from the inner lumen of the scaffold, we did not add hEC-eGFP cells to the perfusion medium in this experiment. A full coverage of endothelial cells on the scaffold surface was detected 24 h after 2 cycles of print-and-culture process, regardless the addition of angiogenic factors to the perfusion medium (Fig. 4E). On post printing day 8, a random overgrowth of endothelium (represented by clumps of endothelial cells) was observed on the scaffold perfused with RPMI-1640 medium without angiogenic factors (Fig. 4F). Excitingly, with the perfusion of angiogenic factors, capillary-like structures started to form at the 4th day post printing, and became more regular-shaped along with the culture process (Fig. 4G–I and Fig. S11D). In addition, we observed sprouting of endothelial cells from the vascular wall under the angiogenic factor perfusion condition at post printing day 18 (Fig. S11E), which later formed a secondary vascular structure projecting toward the culture environment (Figs. S11E and F). Such process highly resembled *in vivo* vasculogenesis [[15], [16], [17]], and the vascular outgrowth formed a lumen by a thin layer of cells, with more endothelial cells distributed around the tip (Fig. S11F), demonstrating the potential of our bioprinting system to facilitate vasculogenesis and angiogenesis among printed cells.

Supplementary data related to this article can be found at https://doi.org/10.1016/j.bioactmat.2022.02.009.

The following is the supplementary data related to this article:

Multimedia component 6 Multimedia component 6

To further explore the angiogenic and vasculogenic ability of our printed artificial blood vessels, we cultured them either on Matrigel matrix (Fig. S12A) or adjacent to an aggregate of smooth muscle cells (aSMCs) (Fig. S13A). Angiogenic projections from the artificial blood vessel were observed within 3 days of Matrigel culture (Fig. S12B), and after 3 weeks of adjacent culture to aSMCs (Figs. S13B and C). These results indicated the potential of our artificial blood vessels to form connections with already existed blood vessel networks after *in vivo* implantation.

### 3.6 Generation of vascularized and contractable cardiac tissues by the bioprinting system

**Fig. 5:**
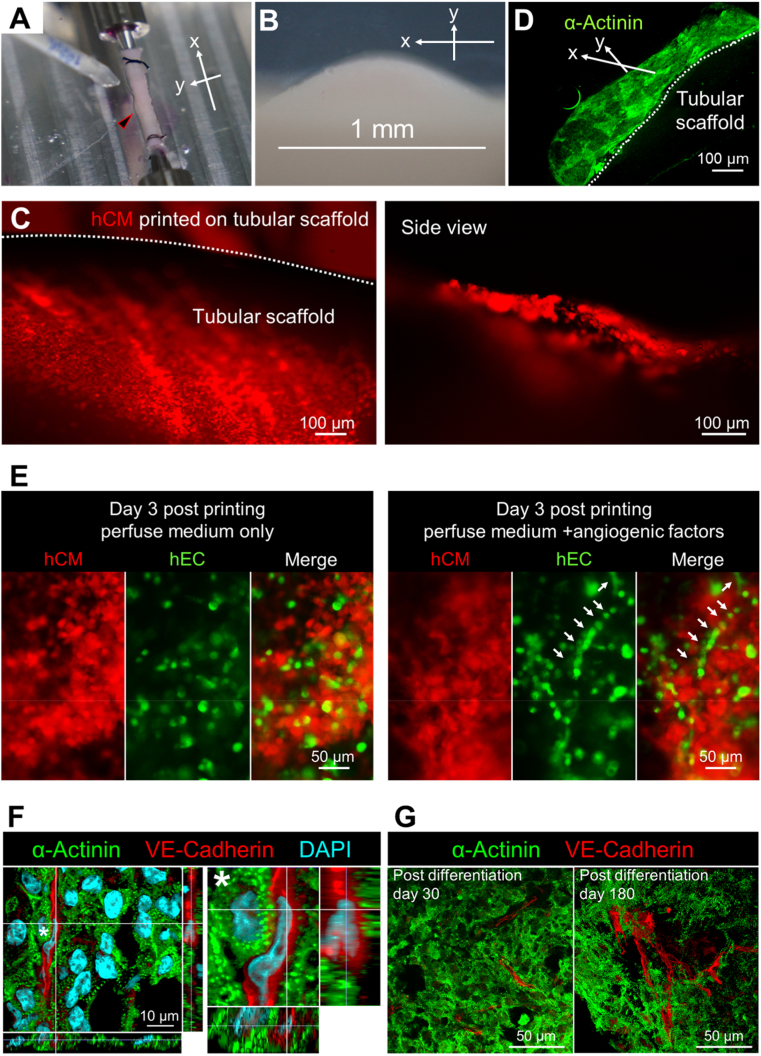
Generation of vascularized cardiac tissues via bioprinting. (**A**) Bioprinting of bioink (H9-differentiated cardiomyocytes) onto a defined spot (indicated by the black arrowhead) of the tubular scaffold. (**B**) Zoom-in image of printed cells in **A**. (**C**) Printed tdTomato-labeled cardiomyocytes form a full-layer on the scaffold and resume beating (see Video S6). (**D**) Cross-section of the tubular scaffold at 2 days post-printing. Cardiac muscular bundle is visualized by immunostaining of sacomeric Actinin (α-Actinin). (**E**) Post-printed hCMEC/d3-eGFP cells (green) and cardiomyocytes-tdTomato (red) resume contraction and form capillary-like structures after cultured in the bioreactor for 3 days with the perfusion of angiogenic factors (see Video S7). White arrows indicate capillary-like structures. hCM, H9-tdTomato differentiated cardiomyocytes. hEC, hCMEC/d3-eGFP. (**F** and **G**) Histological characterizations of the print-and-differentiated vascularized cardiac tissues. Cardiomyocytes (α-Actinin) and endothelial cells (VE-cadherin) are detected in post-differentiation day 30 (**F** and **G**) and day 180 (**G**). Capillary-like structures of the endothelial cells are shown in both sagittal and transverse planes from multi-dimensional Z-slice (**F**), the inter-connected vascular networks are shown in Z-volume (**G**). In **A** to D, x-axis represents the direction in parallel with the tubular scaffold, y-axis represents the direction perpendicular to the tubular scaffold.

Taking advantage of the angiogenesis ability of our bioprinting system, we next evaluated its feasibility in producing complex tissues, such as vascularized cardiac tissues. We first tested whether the functions of cardiomyocytes could be preserved after bioprinting by examining the muscular bundle formation and collective contraction of cardiomyocytes. After printing cardiomyocyte (hCM) bioinks (Fig. S14A) onto the tubular scaffold (Fig. 5A and B), the printed hCMs resumed collective contraction within 48 h under the normal cardiomyocyte culture condition and maintained rhythmic beating for at least 10 days (Fig. 5B and C and Video S6). In addition, the printed hCMs also formed well-organized sacomeric Actinin^+^ muscular bundles (Fig. 5D), resembling the *in vivo* structures of cardiac tissues.

**Fig. 6:**
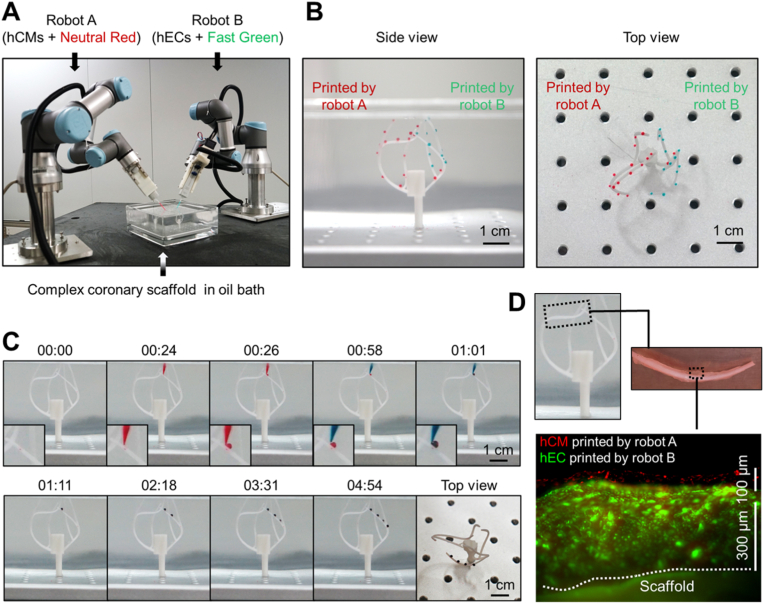
Two-robot platform enables cooperative bioprinting of different bioinks onto complex-shaped scaffolds. (**A**) Setup of the two-robot bioprinting platform. Two 6-DOF robot-based bioprinters are loaded with bioinks containing either Neutral Red-stained hCMs (colored in red) or Fast Green-stained hECs (colored in green) to carry out bioprinting on a vascular scaffold resembling coronary major vessels. (**B**) Side view and top view of the printed bioinks on the scaffold. (**C**) Jointly printing of two bioinks onto the positions of the scaffold. Inset images show the alternative printing of the red-colored and green-colored bioinks to the same positions by the two robots. (**D**) A well-organized hEC-hCM cell layer structure produced by jointly printing of the two robots on one branch of the coronary scaffold. Shown are cells on post-printing day 4.

Supplementary data related to this article can be found at https://doi.org/10.1016/j.bioactmat.2022.02.009.

The following is the supplementary data related to this article:

Multimedia component 7 Multimedia component 7

In order to generate vascularized cardiac tissue, we mixed hCM-tdTomato with hEC-eGFP at 10:1 ratio and printed the cell mixture onto a tubular scaffold (Fig. S14B). Similar to the results mentioned above, the printed cardiomyocytes resumed contraction within 2 days post-printing. With the perfusion of angiogenic factors, capillary-like structures formed by self-organized hEC-eGFP were observed 3 days post-printing (Fig. 5E and Video S7). Moreover, angiogenic perfusion also prolonged the synchronous contraction of cardiac tissue for more than 30 days.

Supplementary data related to this article can be found at https://doi.org/10.1016/j.bioactmat.2022.02.009.

The following is the supplementary data related to this article:

Multimedia component 8 Multimedia component 8

In addition to differentiated cells, we further examined whether our bioprinting system was capable to handle more fragile human embryonic stem cells (hESCs) and support organ generation through stem cell differentiation. We first tested whether our robot bioprinter was suitable for stem cell operations. H9 hESC colonies passaged by the bioprinter for 5 consecutive times still maintained their colonial morphology, with ∼95% cells remained SOX2^+^ and OCT4^+^ (Figs. S15A and B). These hESCs had normal karyotype and proliferation speed, and were comparable to the ones passaged manually (Figs. S15C and D). We therefore prepared the H9 hESCs as bioink and printed them onto a tubular scaffold with 3 print-and-culture cycles and cultured them for 2 days, then carried out cardiovascular differentiation inside the bioreactor by changing culture mediums following a WNT-pathway based cardiovascular differentiation protocol [12] (Fig. S16A). Angiogenic factors were added to the perfusion medium on day 8 post differentiation (day 8 PD), and the first observable cardiac contraction of the printed and differentiated cells appeared on day 12 PD (Video S8). By applying this print-and-differentiation strategy, we fabricated a piece of vascularized cardiac tissue (2 cm in length, 200–500 μm in thickness, and ∼1.256 cm^2^ total area) on the tubular scaffold (Fig. S16B), which maintained contraction for at least 6 months (Video S9). We carried out histological analyses of cardiac tissue on day 30 and day 180 PD, and found no noticeable tissue damage at both time points. In addition, cardiomyocytes were striated with well-organized Z-lines (marked by α-Actinin in Fig. 5F and G), suggesting that the fabricated cardiac tissue had developed and maintained intact myofibril structures, which is the physiological basic for heart contraction [18,19]. Cardiac differentiation from hESCs in the bioreactor also generated VE-Cadherin^+^ endothelial cells, which were deeply buried inside the cardiac muscles (Fig. 5F and G) and formed capillary-like structures with potentially functional lumens (Fig. 5F). During the long-term culture, endothelial cells in the 180-day-PD cardiac tissue exhibited better network-like structures by forming more vascular branches and branching points as comparing to the 30-day-PD cardiac tissue (Fig. 5G and Figs. S16C and D). Such endothelial network highly resembled the structure of coronary plexus [20], which may play a key role in maintaining the vitality and activity of the cardiac tissue during long-term culture. Taken together, these data demonstrated the capability and superiority of our robot bioprinting system in engineering vascularized functional tissues.

Supplementary data related to this article can be found at https://doi.org/10.1016/j.bioactmat.2022.02.009.

The following are the supplementary data related to this article:

Multimedia component 9 Multimedia component 9

Multimedia component 10 Multimedia component 10

### 3.7 Two-robot platform enables flexible and cooperative printing on a complex-shaped coronary scaffold

Unlike the Cartesian bioprinters, the functions of our robot bioprinter can be easily extended by combining multiple robots to finish complicated bioprinting tasks. For a proof of concept, we established a two-robot platform to coordinately print hEC and hCM bioinks onto a complex-shaped blood vessel scaffold, which partially resembled the heart coronary network (Fig. 6A and Fig. S17A). To better visualize the cooperative printing process, we stained cells in each bioprinter with Neutral Red (hCM) or Fast Green (hEC), respectively (Fig. 6A). The two bioprinters were first programmed to print cells onto separate target sites on the scaffold. As shown in Fig. 6B and Video S10, such coordinated operation enabled fast printing of cells on the complex-shaped scaffold to cover all target sites. However, when we restricted the motion of the robotic arms only in the XYZ directions to mimic the movement of Cartesian bioprinters, about 1/3 positions shown in Fig. 6B became unreachable (Fig. S17B). Next, we set the two-robot platform to alternatively print hEC and hCM bioinks onto the same position to mimic the patterned cell type compositions of organs (Fig. 6C and Video S11). Under such settings, we completed a full coverage of alternatively printed hEC-eGFP and hCM-tdTomato bioinks on one branch of the coronary scaffold. The printed cells formed about 0.4 mm thick well-organized layers, with hCMs on the top of hECs (Fig. 6D), indicating that larger and more complex artificial organs could be generated by our bioprinting platform if more cells and robot bioprinters were used and more print-and-culture cycles were applied.

Supplementary data related to this article can be found at https://doi.org/10.1016/j.bioactmat.2022.02.009.

The following are the supplementary data related to this article:

Multimedia component 11 Multimedia component 11

Multimedia component 12 Multimedia component 12

## 4 Discussion

Vascularization and sustained cell survival have been the long-lasting challenges for *in vitro* organ fabrication [1,2,6,8,9]. In this study, we developed a novel bioprinting system with a 6-DOF robot-based bioprinter and a self-designed bioreactor to conquer these problems. By taking advantage of highly flexible robotic arms and a hydrophobic force-mediated cell attachment strategy, we were able to achieve bioprinting on complex-shaped scaffolds with minimum detrimental effects on printed cells. In addition to the culture medium in the tank of the bioreactor, the system also utilized consistently perfused medium through the vascular scaffolds to provide nutrients to printed cells. Thus, both the outer layer and inner layer cells on the vascular scaffolds were able to obtain nutrient supplies. We also demonstrated that the repeated print-and-culture procedure, together with the addition of angiogenic factors to the perfusion medium, enabled vasculogenesis and angiogenesis in printed blood vessels and cardiac tissues. The system therefore demonstrated a feasible way to generate large-scale and functional artificial tissues/organs *in vitro*.

Most of the reported bioprinting methods use self-consolidation artificial biomaterials as the "glue" to attach cells after printing [2,6,7]. Although some biomaterials could degrade after certain period, their presence still hinders the formation of cell-cell connections and impedes nutrient supplies to printed cells [2,21,22]. Here, we creatively employed the hydrophobic force between the oily printing environment and the water-based bioink to warrant the attachment of printed cells to the scaffolds, such strategy not only preserved cell activity to the greatest extent, but also facilitated the formation of blood vascular networks.

In principle, the 6-DOF robot has infinite solutions to reach a destiny coordinate in the space, it therefore could print cells onto any position of a complex-shaped scaffold [10,11]. In the proof-of-concept experiments, we demonstrated the feasibility of using single robot bioprinter or coordinating of multiple robot bioprinters to print cells on complex-shaped vascular scaffold. Such combination of multiple robots into a collaborative printing platform could meet the practical needs for simultaneously printing of multiple cell types, thus to generate complex tissues/organs with patterned cell compositions in a more efficient way.

## CRediT authorship contribution statement

**Zeyu Zhang:** Investigation, Methodology, Data curation, Formal analysis, Visualization, Writing – original draft. **Chenming Wu:** Investigation, Methodology. **Chengkai Dai:** Investigation, Methodology, Software. **Qingqing Shi:** Investigation, Software, Validation. **Guoxin Fang:** Methodology, Software. **Dongfang Xie:** Resources. **Xiangjie Zhao:** Methodology. **Yong-Jin Liu:** Conceptualization, Supervision, Funding acquisition, Writing – review & editing. **Charlie C.L. Wang:** Conceptualization, Supervision, Writing – review & editing. **Xiu-Jie Wang:** Conceptualization, Supervision, Funding acquisition, Writing – review & editing.

## Declaration of competing interest

None.
