# Supplementary material for: A multi-axis robot-based bioprinting system supporting natural cell function preservation and cardiac tissue fabrication
Source: Bioact Mater. 2022 Feb 19;18:138–50. doi: 10.1016/j.bioactmat.2022.02.009 (PMC8961309; doi:10.1016/j.bioactmat.2022.02.009)
Supplement: Multimedia component 1 [file mmc1.docx]

**Supplementary Materials**

**A multi-axis robot-based bioprinting system supporting natural cell function preservation and cardiac tissue fabrication**

Zeyu Zhang^1,5,6^, Chenming Wu^2,6^, Chengkai Dai^4,6^, Qingqing Shi^1,6^, Guoxin Fang^3,4^, Dongfang Xie^1^, Xiangjie Zhao^1,5^, Yong-Jin Liu^2,^*, Charlie C.L. Wang^3,4,^* & Xiu-Jie Wang^1,5,^*

^1^ Institute of Genetics and Developmental Biology, Innovation Academy of Seed Design, Chinese Academy of Sciences, Beijing 100101, China

^2^ Beijing National Research Center for Information Science and Technology, Department of Computer Science and Technology, Tsinghua University, Beijing 100084, China

^3^ Department of Mechanical, Aerospace and Civil Engineering, The University of Manchester, Manchester M20 4BX, UK

^4^ Faculty of Industrial Design Engineering, Delft University of Technology, Delft 2628, The Netherlands

^5^ University of Chinese Academy of Sciences, Beijing 100049, China

^6^ These authors contributed equally: Zeyu Zhang, Chenming Wu, Chengkai Dai, Qingqing Shi

**Correspondence:**

Xiu-Jie Wang: xjwang@genetics.ac.cn

Charlie C.L. Wang: [changling.wang@manchester.ac.uk](mailto:changling.wang@manchester.ac.uk)

Yong-Jin Liu: liuyongjin@tsinghua.edu.cn

**Materials and Methods**

**Printing trajectory demonstration with the 3D human heart resin model**

To draw a coronary network on the 3D human heart resin model, a 3D printed resin fixture holding a red marker pen was installed onto the end-effector of the UR3 robotic arm (Fig. S5A-C). Representative coordinates for drawing the coronary network on the human heart model were extracted and smoothened as the moving paths for the robotic arm. We next calibrated the robotic arm to ensure sufficient touching between the tip of the marker pen and the surface of the heart model, then we programmed the robotic arm to move along the path and draw a coronary network on the 3D heart model.

**Lentivirus production and infection of hCMEC/d3 cells**

To generate stable hCMEC/d3-eGFP cell strain, we first generated and amplified eGFP labeled lentiviruses in HEK293T cells. Lentiviruses were first packaged by co-transfection of pLL3.7 vectors containing CMV-eGFP reporters (Addgene #11795) with packaging vectors psPAX.2 and pMD2.G (Addgene #12260 and #12259) into HEK293T cells using Lipofectamine LTX (Gibco #15338100). After transfection, the cells were cultured in DMEM + 10% FBS for 72 hours. To harvest lentiviruses, transfected HEK293T cells were collected in an Amicon Ultra tube (Merk #UFC910096) and centrifuged at 2475 *g* for 30 min at 4°C. The lentiviruses were titrated to 5 × 10^8^ infection units/ml using Lenti-X GoStix kit (Takara #631244), then added to the culture medium (RPMI-1640 + 10% FBS) of hCMEC/d3 cells to achieve a multiplicity of infection (MOI, average number of lentiviral particles per cell) of 10. hCMEC/d3 cells were cultured in this lentivirus-containing culture medium for 3 days and then washed with 1 × PBS twice, digested with 0.25% Trypsin and suspended in RPMI-1640 medium. To enrich eGFP+ cells, the cell suspension was subjected to fluorescent-activated cells sorting (FACS) using a FACSAriaII system (BD Biosciences). The selected cells were cultured for 7 days then FACS sorted again to obtain pure eGFP^+^ hCMEC/d3 cells.

**Generation of H9-tdTomato cells**

To establish an AAVS1-targeted H9-tdTomato knock-in cell strain, we electroporated AAVS-tdTomato vector (Addgene #159275) and AAVS1-T2-CRIPR-in-pX330 vector (Addgene #72833) into H9 hESCs using Lonza P3 kit (Lonza #V4XP-3024) following the Optimization Guideline on the company’s website (*https://knowledge.lonza.com/cell?id=601&search=ESC*). In brief, TeSR-E8 cultured H9 colonies were detached and dissociated by Accutase, then 1 × 10^6^ H9 cells were suspended in 82 μl prewarmed Nucleofector Solution (Lonza #V4XP-3024). Vector mixture (5 μg DNA in 10 μl ddH_2_O) was added to 18 μl Supplement (Lonza #V4XP-3024), which was then mixed with the cell suspension and transferred to a Nucleocuvette vessel (100 μl volume in Lonza #V4XP-3024 kit). Cells were electroporated using a 4D-Nucleofector X Unit (Lonza) with the CB-150 program setting, then suspended in prewarmed StemFlex medium (Gibco #A3349401) and cultured in 1% Matrigel coated 6-well plates. StemFlex medium was refreshed daily, and 0.5 μg/ml Puromycin (Sigma #P7255-10) was added to the culture medium on the 3^rd^ and 4^th^ days. On the 7^th^ day, Puromycin-resistant tdTomato^+^ H9 colonies were manually picked and cultured in TeSR-E8 medium without Puromycin.

**Antibodies used in immunohistochemical analysis**

Antibodies used in this study are: Anti-alpha Tubulin (Abcam #ab7291, 1:1000), Phospho-H2A.X-Ser139 (CST #2577, 1:1000), Anti-53BP1 (Abcam #ab175933, 1:500), Anti-Sarcomeric Alpha Actinin (Abcam #ab9465, 1:500), Anti-Connexin 43 (Abcam #ab11370, 1:500), Anti-VE Cadherin (Abcam #ab205336, 1:300), Anti-E Cadherin (Abcam #ab11512, 1:500), Anti-Sox2-Alexa Fluro 488 (Abcam #ab195358, 1:500), Anti-Oct4-Alexa Fluro 647 (Abcam #ab196585, 1:500), Goat Secondary Antibody to Mouse-Alexa Fluro 488 (Abcam #150113, 1:1000), Goat Secondary Antibody to Rabbit-Alexa Fluro 594 (Abcam #ab150080, 1:1000) , and Goat Secondary Antibody to Rat-Alexa Fluro 594 (Abcam #ab150160, 1:1000).

**Characterization of robot passaged hESCs**

To evaluate whether bioprinting would have any negative impacts on the pluripotency and genome stability of hESCs, we detached H9 colonies using ReLeSR and plated them by robotic bioprinting for 5 consecutive passages. In each passage, H9 colonies were maintained in TesR-E8 for 4 days, and in the 5^th^ passage, cells were subjected to downstream analyses when colonies reaching 80% confluency.

To analyze the pluripotency maintenance of printed hESCs, H9 colonies were dissociated, fixed, permeabilized and blocked as previously described, then antibody-labeled by incubating with Anti-SOX2-Alexa 488 (Abcam #ab195358, 1:500) and Anti-OCT4-Alexa 647 (Abcam #ab196585, 1:100) diluted in 0.1% Triton X-100 + 2% BSA for 30 min at RT. Cells were washed by 1 × PBS for 3 times, resuspended in 1% BSA (1 × PBS) and analyzed by FACSAriaII. H9 cells incubated with rabbit IgG-Alexa 488 (Abcam #ab199091, 1:500) and rabbit IgG-Alexa 647 (Abcam #ab199093, 1:500) were used as the isotope control of FACS analysis.

To analyze the karyotype of printed hESCs, demecolcine (50 ng/ml, Enzo #ALX-430-033) was added to H9 culture medium and incubated for 8 hours at 37°C, then cells were dissociated by Accutase, collected and treated with 0.075 M KCl for 30 min at 37°C. Cells were washed with methanol + acetic acid (v/v = 3:1) for 3 times and incubated in methanol + acetic acid (v/v = 3:1) overnight at 4 °C. After overnight incubation, the cell solution was hanging dropped onto ice cold slides using a Pasteur pipette, oven dried at 60°C, stained with Giemsa (Solarbio #G8220) for 5 min at RT and washed by ddH_2_O. Chromosome G bands were visualized using an Olympus microscope and analyzed using VideoTesT-Karyo (v. 3.1).

**Proliferation and survival test of robotic bioprinted cells under mineral oil incubation**

To evaluate whether the mineral oil incubation process would bring any detrimental effects on bioprinted cells, we measured the proliferation and survival rates of manually seeded cells and robotic bioprinted cells after mineral oil incubation. In brief, we prepared endothelial cell bioinks by suspending hCMEC/d3 cells in RMPI-1640 medium consisting of 10% FBS at a concentration of 5000 cells/μl, then either manually seeded the bioink onto a 96-well plate (1 μl/well) filled with normal cell culture medium (RPMI-1640 + 10% FBS) or robotic bioprinted the bioink onto a 96-well plate (1 μl/well) filled with mineral oil (1 μl/well). Both 96-well plates were then transferred into a 37°C incubator, and the mineral oil was replaced by normal cell culture medium (RPMI-1640 + 10% FBS) 4 hours later. Both 96-well plates were incubated at 37°C for 48 hours, then cells were stained with the Live-Dead Cell Staining Solution (Solarbio #CA1630) and recorded by a Leica fluorescence microscope. We next digested cells using 0.25% Trypsin and counted cell numbers by Countess II FL.

**Bioprinting on plane Petri dish and 3D vascular scaffold**

To print bioink containing green florescent hCMEC/d3 cells into the shape of number “10” or letters “IGDB” on Petri dish, coordinates outlined “10” or “IGDB” were sampled and smoothened as the moving paths of the robot. To print bioink at defined positions on the 3D vascular scaffold, printing positions were sampled into coordinates, trajectory of the robot movement were computed by inverse kinematics. Before the bioprinting process, we carried out calibration for the robotic arm to adjust the linear distance between the tip of the ejector and the printing surface to be 1 mm, bioink printed within this distance would attach to the surface of the Petri dish or scaffold. For discrete printing, the robotic arm was programmed to stop at each printing position for 1 second to wait for the ejection to complete, whereas for the continuous printing, the robotic arm was programmed to move along the printing path at a speed of 1 mm/s during the printing process without any pausing.

Bioink was prepared by suspending hCMEC/d3-eGFP cells in RMPI-1640 + 10% FBS (5 × 10^6^ cells/ml), and was printed as 2 μl per ejection for printing discrete “10” and “IGDB”, 4 μl per ejection for printing continuous “10” on Petri dish, and 1 μl per ejection for printing discrete points on the 3D vascular scaffold.

**Cell perfusion test in the bioreactor**

For the endothelial cell perfusion test (Fig. 4B and C, and S10F), we prepared the cell perfusion medium by suspending hCMEC/d3-eGFP cells in RPMI-1640 medium consisting of 10% FBS, 1 × antibiotic-antimycotic, 1% Matrigel, and 10 mM HEPES (Gibco #15630080) at a concentration of 1 × 10^6^ cells/ml. The buffer medium used in the bioprinting environment setup stage was replaced by mineral oil for medium in the tank, and by cell perfusion medium for medium in the circulation loop. Cell perfusion medium in the circulation loop was continuously perfused through the tubular scaffold for 24 hours at a speed of 1 ml/min. Cross sections of the scaffold were visualized and imaged under a fluorescence microscope (Leica DMI 3000B).

**Bioprinting of artificial blood vessels**

To fabricate artificial blood vessels via bioprinting (Fig. 4D), we first prepared endothelial bioink by suspending hCMEC/d3-eGFP cells in cold endothelial medium (RPMI-1640 medium supplemented with 10% FBS, 1% Matrigel, 1 × GlutaMax, 1 × NEAA and 10 mM HEPES) at a concentration of 5 × 10^6^ cells/ml, and printed the bioink onto the tubular scaffold on day 1 and day 2 as described above (2 print-and-culture cycles). During the post-printing culture process, mineral oil in the tank was replaced with the endothelial medium supplemented with 1 × antibiotic-antimycotic, and the tubular scaffold was perfused with RPMI-1640 medium supplemented with 2% B27, 1% Matrigel and 10 mM HEPES with or without the addition of the following angiogenic factors: bFGF (100 ng/ml, PeproTech #100-18B-100), hVEGF (100 ng/ml, PeproTech #100-20-10), hEGF (100 ng/ml, PeproTech #AF-100-15-500), hIGF-I (200 ng/ml, PeproTech #100-11-20), Heparin (1 U/ml, Sigma #H3149-10KU) and ascorbic acid (50 μg/ml, Sigma #A8960).

**Bioprinting of vascularized cardiac tissue**

To fabricate cardiac clump via bioprinting (Fig. 5B), we prepared cardiac bioink by suspending H9-differentiated cardiomyocytes in Cardiomyocyte Support Medium supplemented with 1% Matrigel and 2 μM Y-27632 at a concentration of 5 × 10^6^ cells/ml. We repeatedly printed 1 μl bioink to a particular location on the scaffold for 4 time (with 20 min interval between each printing). The post-printed cardiomyocytes formed a cell clump on the scaffold. We then cultured and perfused the scaffold with the cardiac medium (RPMI-1640 medium supplemented with 2% B27, 1% Matrigel, 10 mM HEPES and 1 × antibiotic-antimycotic) for 2 days, and then examined the cardiomyocyte clump with immunohistological analysis. To print a layer of cardiomyocytes fully covering the tubular scaffold (Fig. 5C), we prepared red fluorescent cardiac bioink by suspending H9-tdTomato-differentiated cardiomyocytes in Cardiomyocyte Support Medium supplemented with 1% Matrigel and 2 μM Y-27632 at a concentration of 5 × 10^6^ cells/ml, and printed this bioink on day 1 and day 2 as described above (2 print-and-culture cycles). Cardiac tissue covering the tubular scaffold was post-printing cultured and perfused with the same cardiac medium used for cardiomyocyte clump.

To fabricate vascularized cardiac tissue (Fig. 5E), we prepared bioink mixture of cardiomyocyte-tdTomato and hCMEC/d3-eGFP cells (10:1) in Cardiomyocyte Support Medium supplemented with 1% Matrigel and 2 μM Y-27632 at a concentration of 6 × 10^6^ total cells/ml, and printed the bioink onto the tubular scaffold on day 1 and day 2 as described above (2 print-and-culture cycles). During the post-printing culture process, we switched the mineral oil in the tank to the cardiac medium supplemented with 1 × GlutaMax and 1 × NEAA, and perfused the scaffold with the cardiac medium with or without supplementing the following angiogenic factors: bFGF (100 ng/ml, PeproTech #100-18B-100), hVEGF (100 ng/ml, PeproTech #100-20-10), hEGF (100 ng/ml, PeproTech #AF-100-15-500), hIGF-I (200 ng/ml, PeproTech #100-11-20), Heparin (1 U/ml, Sigma #H3149-10KU) and ascorbic acid (50 μg/ml, Sigma #A8960).

**Bioprinting of human embryonic stem cells and post-printing differentiation induction**

For the bioprinting of hESCs, we prepared hESC bioink by suspending Accutase-dissociated H9 cells in the StemFlex medium supplemented with 1% Matrigel at a concentration of 5 × 10^6^ cells/ml and printed the bioink onto a tubular scaffold on day 1, day 2 and day 3 as described above (3 print-and-culture cycles). We then cultured and perfused the H9-covered scaffold with the StemFlex medium supplemented with 1 × antibiotic-antimycotic for 48 hours (day 4 and 5). On day 6, we initiated cardiac differentiation using the above mentioned Wnt pathway-based method by changing the medium in the bioreactor to the differentiation medium (RPMI-1640 medium supplemented with 2% B27 minus insulin, 1% Matrigel, 10 mM HEPES and 1 × antibiotic-antimycotic) + 8 μM CHIR-99021 (from day 6 to day 7), and to differentiation medium + 2 μM Wnt-C59 (from day 8 to day 9), then to cardiac medium thereafter. The scaffold was perfused with the TeSR-E8 medium from day 1 to day 5, then changed to differentiation medium from day 6 to day 9, then changed to cardiac medium from day 10 to day 12, and changed to cardiac medium supplemented with angiogenic factors (100 ng/ml bFGF, 100 ng/ml hVEGF, 100 ng/ml hEGF, 200 ng/ml hIGF-I, 1 U/ml Heparin and 50 μg/ml ascorbic acid) from day 13 and thereafter. Started from the 13^th^ day, the culture medium in the bioreactor and circulation loop was refreshed every 7 days.

**Angiogenesis assay**

For the angiogenesis assay using endothelial cell bioink (Fig. 2C), Matrigel was diluted in cold RPMI-1640 basal medium (1:3) and incubated at 37°C overnight to form gel matrix in a 96-well plate. The hCMEC/d3-eGFP cell bioink was prepared as described above and either manually seeded or printed onto the Matrigel matrix, then cultured at 37°C with 5% CO_2_ for 24 hours. The numbers of tubule branch points within each well were counted 24 hours after cell seeding/printing. The capillary-like tubule structures formed by endothelial cells on the Matrigel matrix were imaged using a fluorescent microscope (Leica DMI 3000B).

For the angiogenesis assay of bioprinted artificial blood vessels, bioink of hCMEC/d3-eGFP cells was repeatedly printed onto tubular scaffolds on day 1 and day 2 as described above (2 print-and-culture cycles). On day 5, scaffolds were disassembled from the bioreactor for downstream analyses. To test the angiogenic potential of the artificial blood vessel with the support of Matrigel matrix (experiment settings in Fig. S12), Matrigel diluted in cold RPMI-1640 (1:3) was jellified into matrix at 37°C overnight on a Petri dish, the scaffold was then directly placed onto the matrix and cultured with RPMI-1640 + 10% FBS + 1 × GlutaMax + 1 × NEAA + 1 × antibiotic-antimycotic for 3 days without changing the culture medium. Endothelial sprout was monitored daily under a microscope (Leica). To test the angiogenic potential of the artificial blood vessel toward smooth muscle tissues (experiment settings in Fig. S13), MOVAS cells were cultured to 100% confluency to form a cell sheet then entirely detached by Dispase (StemCell #07913). The MOVAS sheet was transferred to an Ultralow Attachment plate with a wide opening pipette tip and was 3D-cultured in DMEM + 10% FBS (37°C with 5% CO_2_) for 48 hours to form an aggregate. The MOVAS aggregate was placed adjacent to the printed tubular scaffold, and they were jointly cultured for over 30 days in the Ultralow Attachment plate with RPMI-1640 + 10% FBS + 1 × GlutaMax + 1 × NEAA + 1 × antibiotic-antimycotic. The medium was refreshed every 3 days. The Ultralow Attachment plate was tilted 30° in the bioreactor to allow directly contact of the MOVAS aggregate with the artificial blood vessel. Sprout of the hCMEC/d3-eGFP cells from the tubular scaffold was monitored daily under a fluorescence microscope (Leica DMI 3000B).

**Supplementary Figures**

**
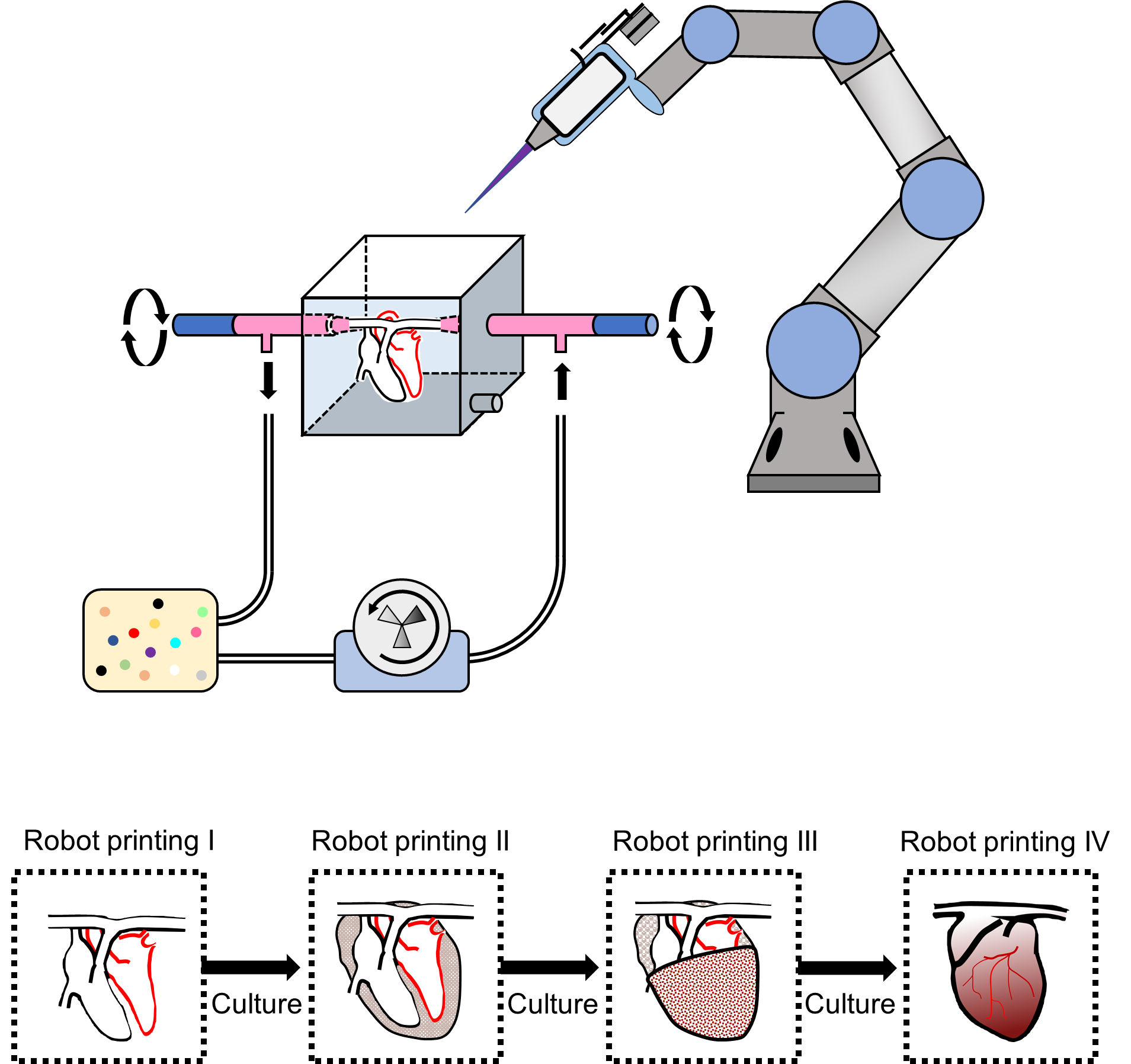
**

**Fig. S1. A carton summary of the self-developed bioprinting platform and the repeated print-and-culture strategy.**

The bioprinting platform developed in this study consists of a 6-DOF robot-based bioprinter and a self-designed bioreactor, which accommodates both the biomaterial-free cell printing and post-printing cell/tissue culture process (upper panel). Facilitated by the biocompatibility and flexibility of the bioprinter, cells can be printed onto complex tridimensional vascular scaffolds from any directions without compromising their biological functions. With such a system, fabrication of large-scale complex organs may be accomplished by applying a repeated print-and-culture strategy (lower panel), which, instead of tempting to finish the bioprinting in a single run, divides the fabrication into several rounds of printing coupled with post-printing cell/tissue cultures. Cells printed at each round are cultured for sufficient time intervals to allow the formation of cell-cell contact and blood vessel networks, which are the prerequisites for generating viable and functional large organs.


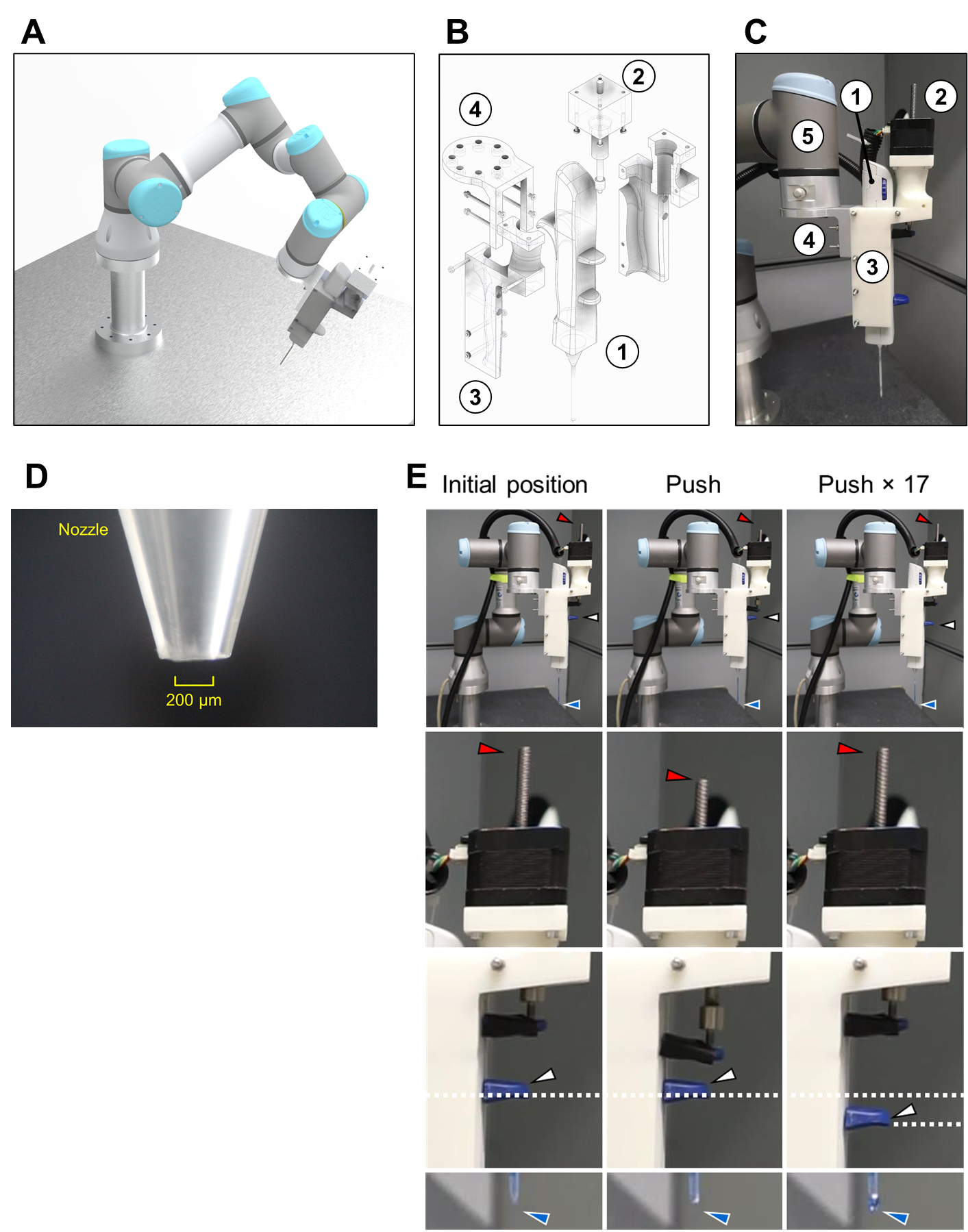


**Fig. S2. Conversion of the highly programmable and flexible 6-DOF robotic arm into a bioprinter.**

(**A**) Three-dimensional model of the assembled bioprinter. (**B**) Design of a fixture to hold and mount the Multipette onto the end-effector of the 6-DOF robotic arm. (**C**) Picture of the assembled bioprinter. The robotic arm serves as the printer arm, the Multipette serves as the cell ejector, and the Mutipette tip serves as the nozzle. (**D**) Enlarged view of the nozzle tip. In **B** to **C**, ① Eppendorf Multipette; ② stepper motor; ③ fixture for holding Multipette; ④ fixture for mounting Multipette to the robotic arm; ⑤ end-effector of the 6-DOF robotic arm. (**E**) Working mechanism of the stepper motor-Multipette-based bioprinter. Photos in the left column show the initial position of the stepper motor and the plugger of the Multipette, photos in the middle column show one single push of the stepper motor with 1 μl Trypan Blue liquid propelled from the tip, photos in the right column show the moving distance of the Multipette’s plugger after 17 consecutive pushes and the Trypan Blue liquid drop at the tip of the nozzle. Rows 2-4 are the zoom-in pictures for positions of the stepper motor (indicated by red arrowheads), the plugger of the Multipette (indicated by white arrowheads), and the bioink drop (with Trypan Blue for visualization, indicated by blue arrowheads) in pictures in the top row. See also Video S1.


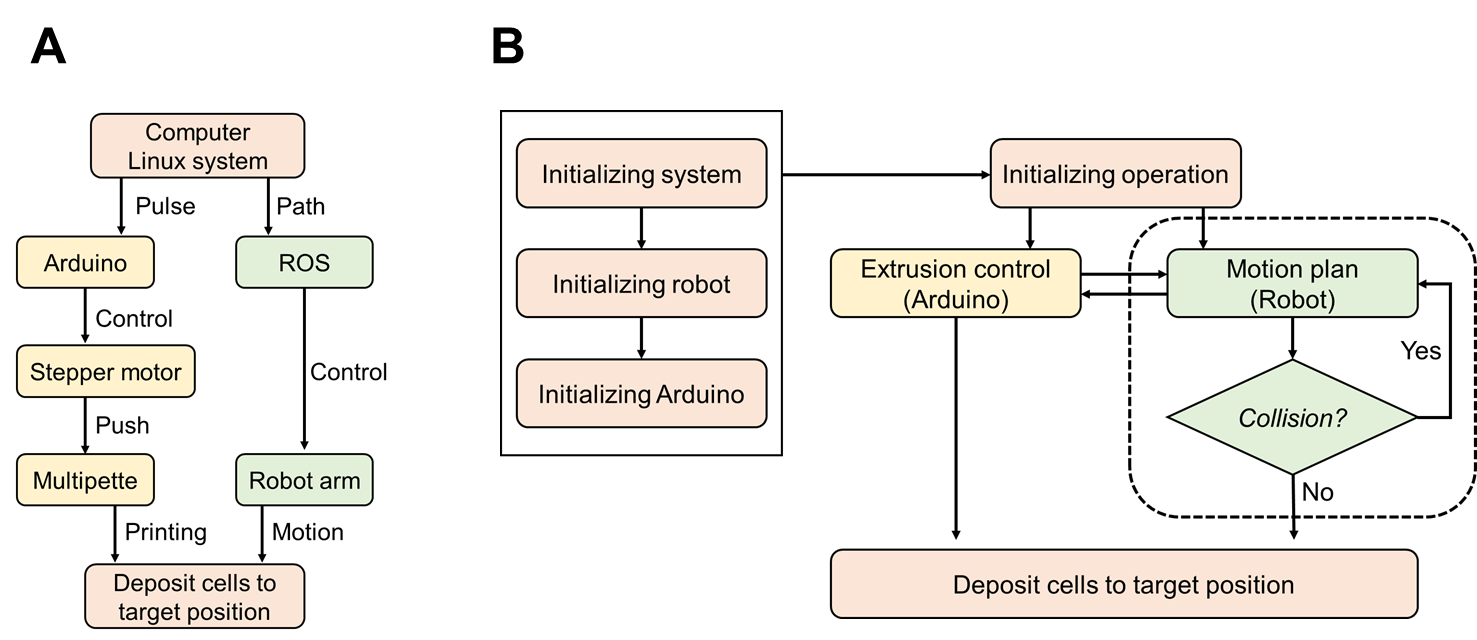


**Fig. S3. Operation program of the 6-DOF robot-based bioprinter.**

(**A**) Software control system of the bioprinter. (**B**) A general operation procedure of the bioprinter.


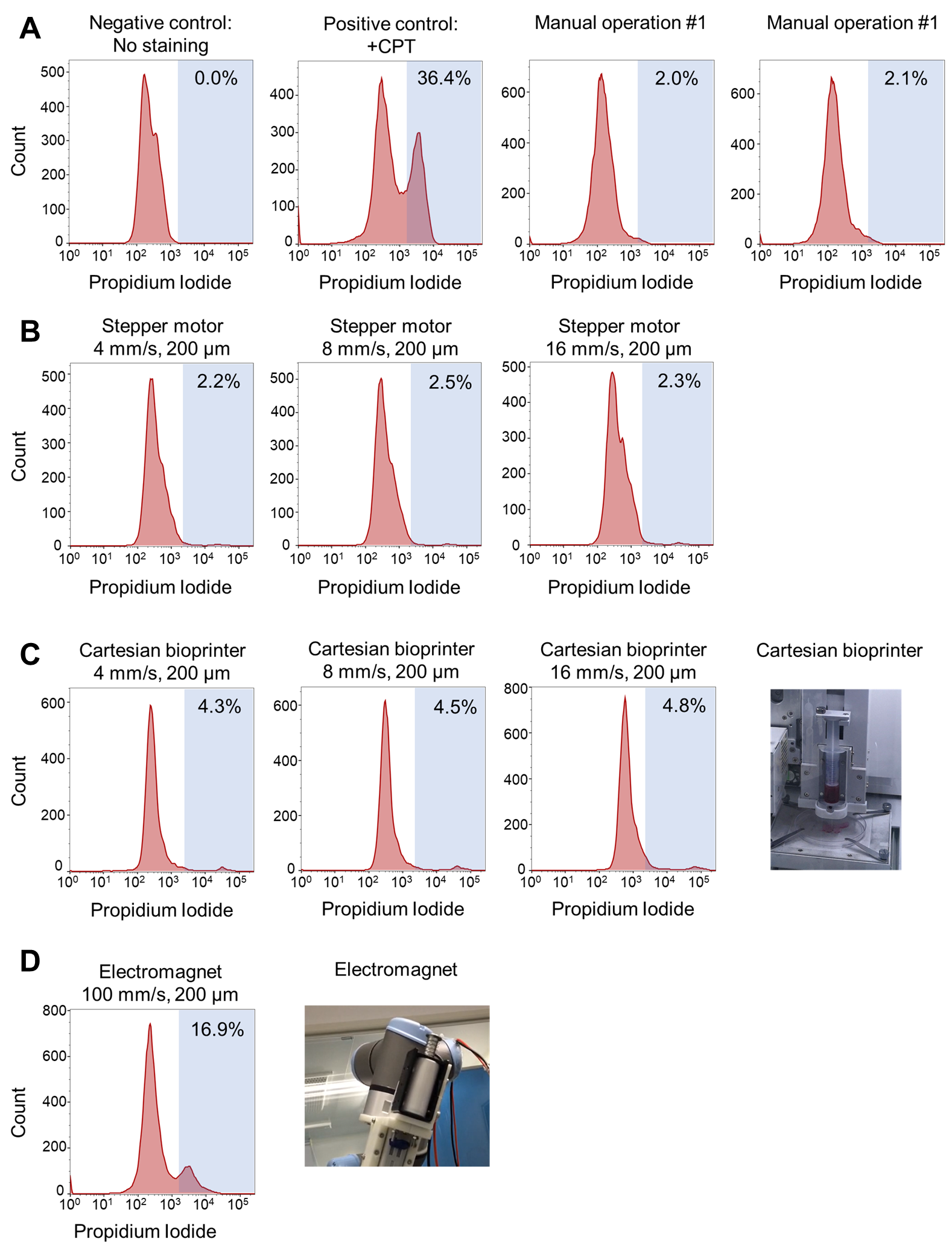


**Fig. S4. Comparison of cell damages caused by different bioprinting systems.**

FACS analysis of propidium iodide (PI) stained hCMEC/d3 cells transferred by manual operation (**A**), 6-DOF robot-based bioprinter (**B**), extrusion-based Cartesian bioprinter (**C**) and electromagnet cell ejector (**D**). Cells with membrane damages are detected by PI staining. hCMEC/d3 cells without PI staining are used as negative control and hCMEC/d3 cells treated with 1 μM Camptothecin (CPT) for 24 hours are used as positive control in the FASC analysis.


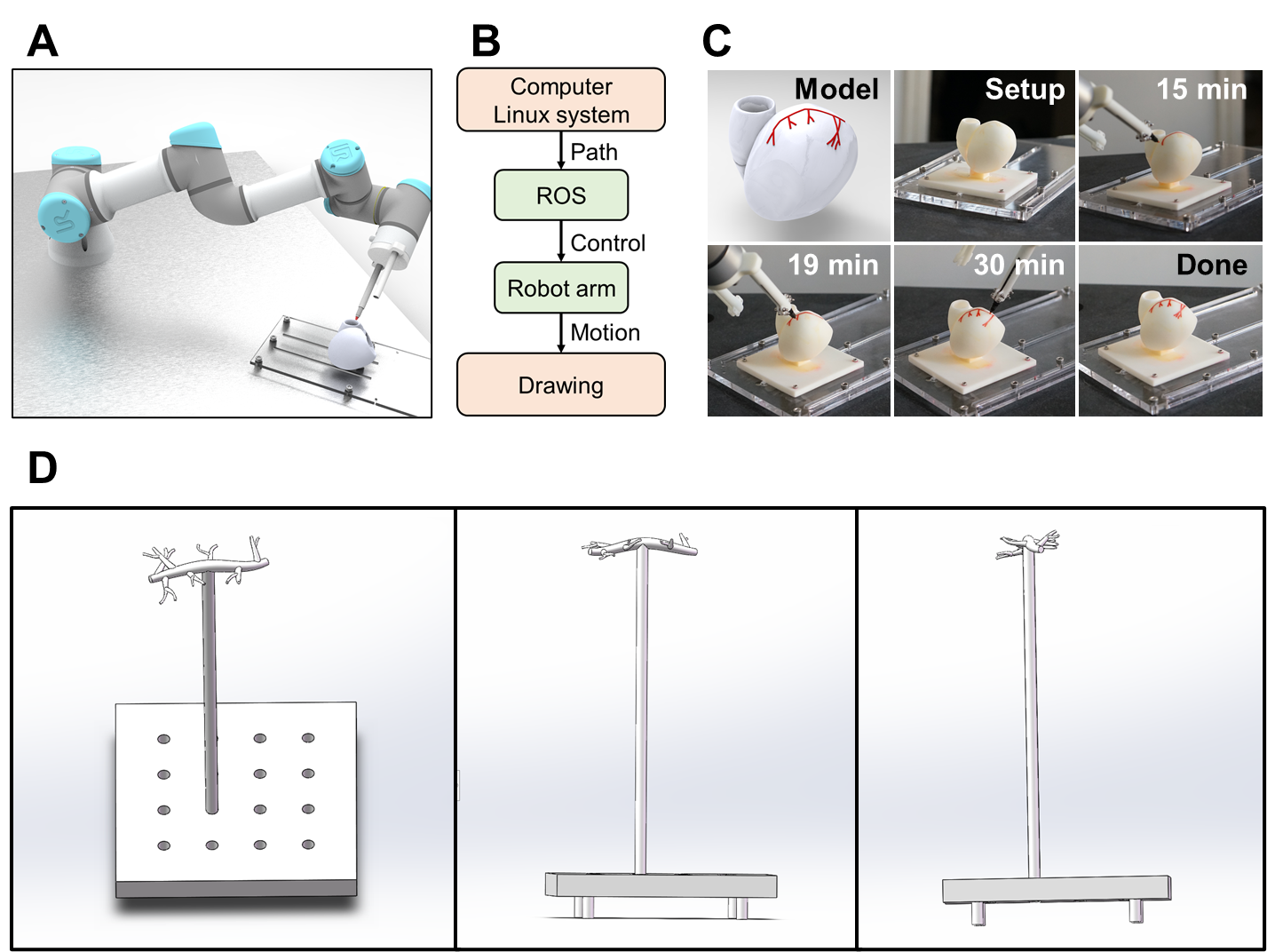


**Fig. S5. Demonstration of the flexibility and controllability of the 6-DOF robot.**

(**A**) Three-dimensional model for the installation of a marker pen to the end-effector of the robotic arm to enable its drawing function. (**B**) Operation procedure of the robotic arm in the drawing experiment. (**C**) Photo recordings of the blood vessel drawing experiment. See also Video S4. (**D**) Three-dimensional model of a complex vascular scaffold.


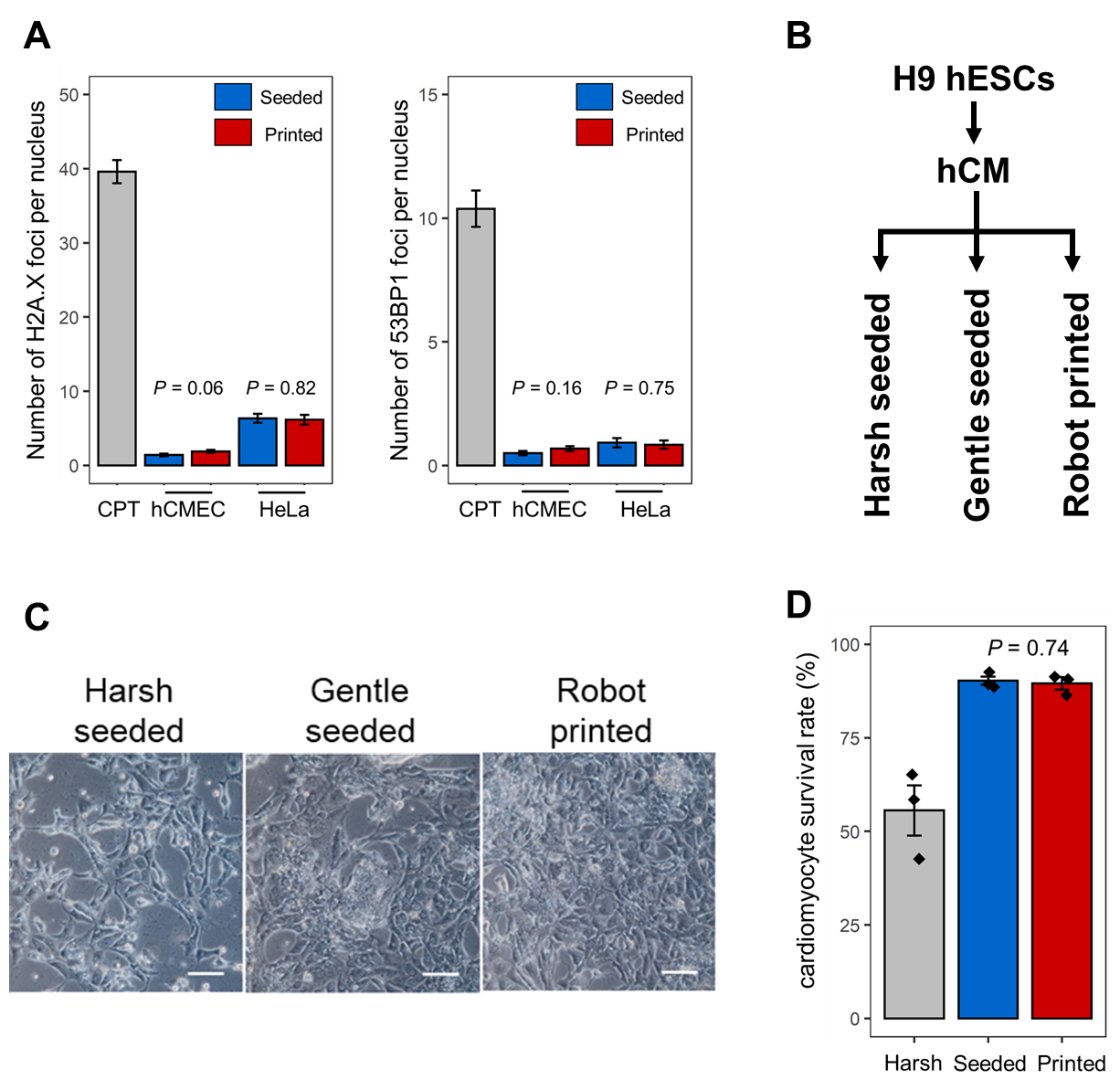


**Fig. S6. The 6-DOF robot bioprinter causes no extra damages to genomic DNA and preserves cell viability.**

(**A**) Statistical analysis of DNA damage foci among manually seeded or printed cells. CPT, hCMEC/d3 cells treated with 1 μM Camptothecin (CPT) for 24 hours to serve as the positive control for DNA damage. n = 50 cells in each group (10 cells per replicate from 5 seeding/printing replicates). (**B**) Experimental setup for cardiomyocyte damage analysis after printing or manual operations. Harsh seeded cardiomyocytes are those harshly pipetted for 10 times before plating, which can cause significant cell death. (**C**) Morphology of human cardiomyocytes plated by manual operation or robot printing. (**D**) Survival statistics of human cardiomyocytes plated by manual operation or robot printing. Scale bars, 50 μm. Error bars, standard error of the mean of 3 replicates, *P*-values are calculated by Student’s *t*-test.

**
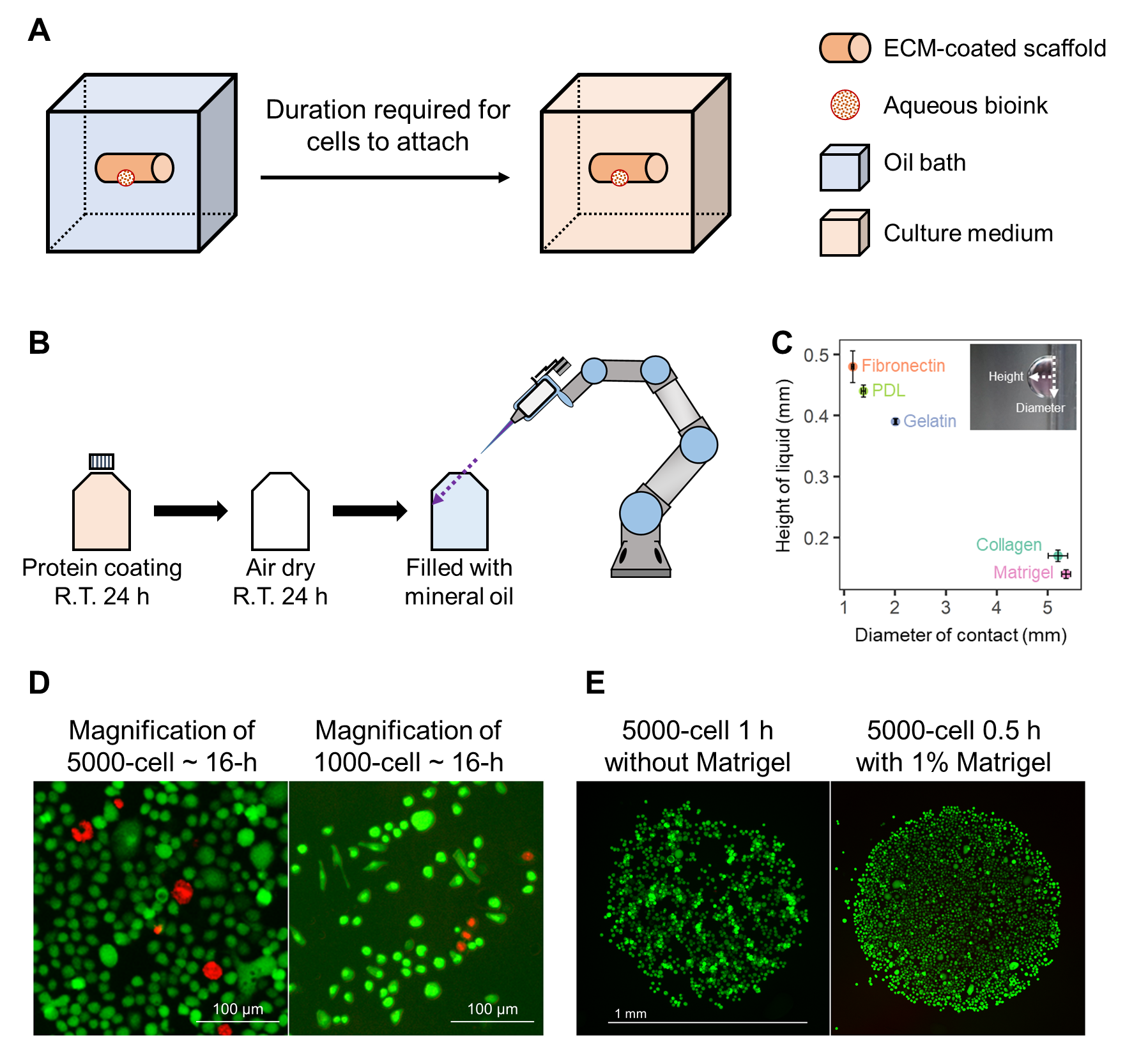
**

**Fig. S7. Oil bath maintains aqueous bioink adherent and promotes cell attachment.**

(**A**) A carton demonstrating the biomaterial-free printing strategy which uses oil bath to facilitate cells to attach to any directional surface. In the mineral oil bath, the propel force from the hydrophobic oil and the pre-coating of extracellular matrix (ECM) protein on the scaffold both help the printed aqueous bioink droplets (containing cells) to attach to the scaffold. Cells will firmly attach to the ECM-coating after incubating for certain period, after which replacing the oil with culture medium to provide long-term culture environment will not disperse cells. (**B**) Schematic diagram showing the experimental design for testing the hydrophilic effect of different ECM coating under oil bath. (**C**) Effects of different ECMs on maintaining the round shape of aqueous bioink droplets. Inset shows the measurement parameters of the droplet shape. **(D)** Enlarged regions of Fig. 3B showing dead cells. (**E**) Supplementing 1% Matrigel to the bioink facilitates cell attachment and the formation of cell monolayer. Error bars in (**C**) are standard error of the mean.


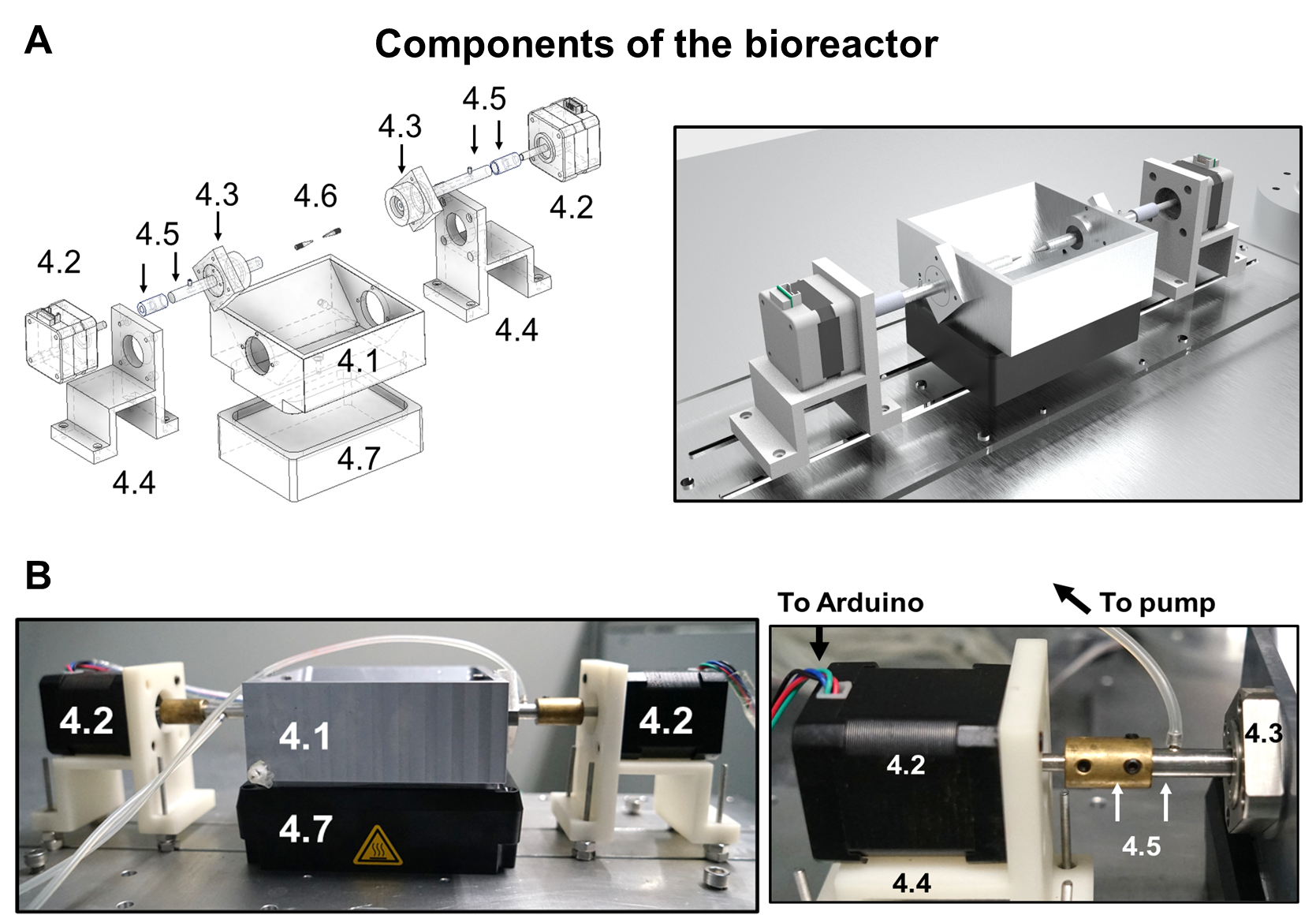


**Fig. S8. Composition and assembly of the bioreactor.**

Components (**A**) and assembly (**B**) of the bioreactor. 4.1, tank; 4.2, rotation motors; 4.3, leak-proof bearing; 4.4, 3D printed shelves; 4.5, hollow rods; 4.6, needles; 4.7, heater.


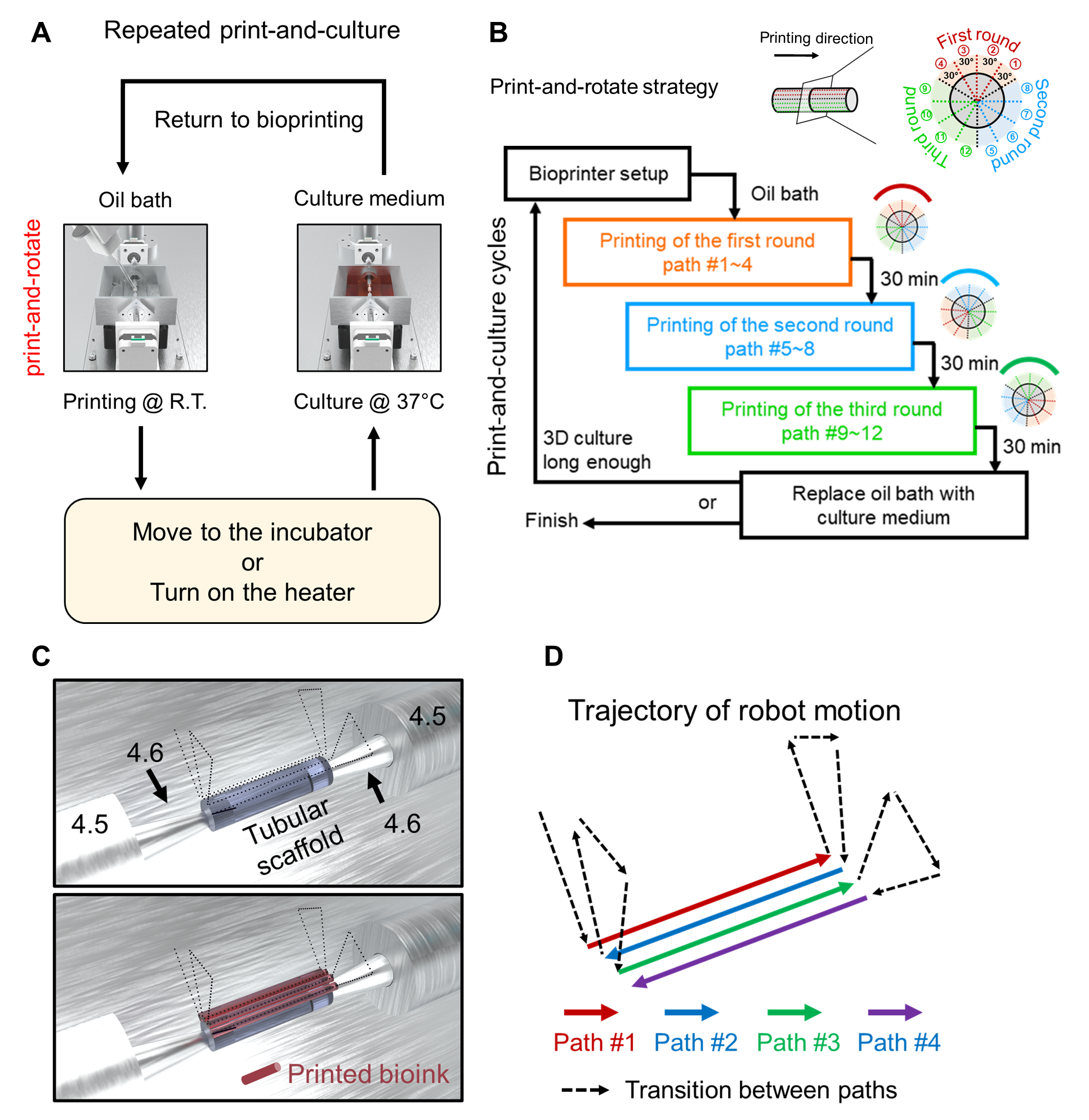


**Fig. S9. Illustration of the repeated print-and-culture bioprinting strategy and printing trajectory along tubular scaffolds.**

**(A)** Schematic workflow of the repeated print-and-culture strategy. (**B**) The print-and-rotate procedure for printing cells on a tubular scaffold. The tubular scaffold is first divided into 3 equal sectors (120° each), and each sector is further divided into 4 equal paths (30° each, numbered 1-12, upper panel). At each print round, cells are printed along the 4 paths, cultured for 30 min in the oil bath, then rotate the rod for 120° to carry out the second round printing, and repeat until finishing all printing paths (bottom panel). (**C**) Three-dimensional modeling of the in-bioreactor tubular scaffold and the robot movement path (dashed lines in **C**). (**D**) The abstracted robot movement trajectories in (**C**). Black dashed lines are transition paths for the robot to avoid collision of the printer tip onto the scaffold while changing print paths.


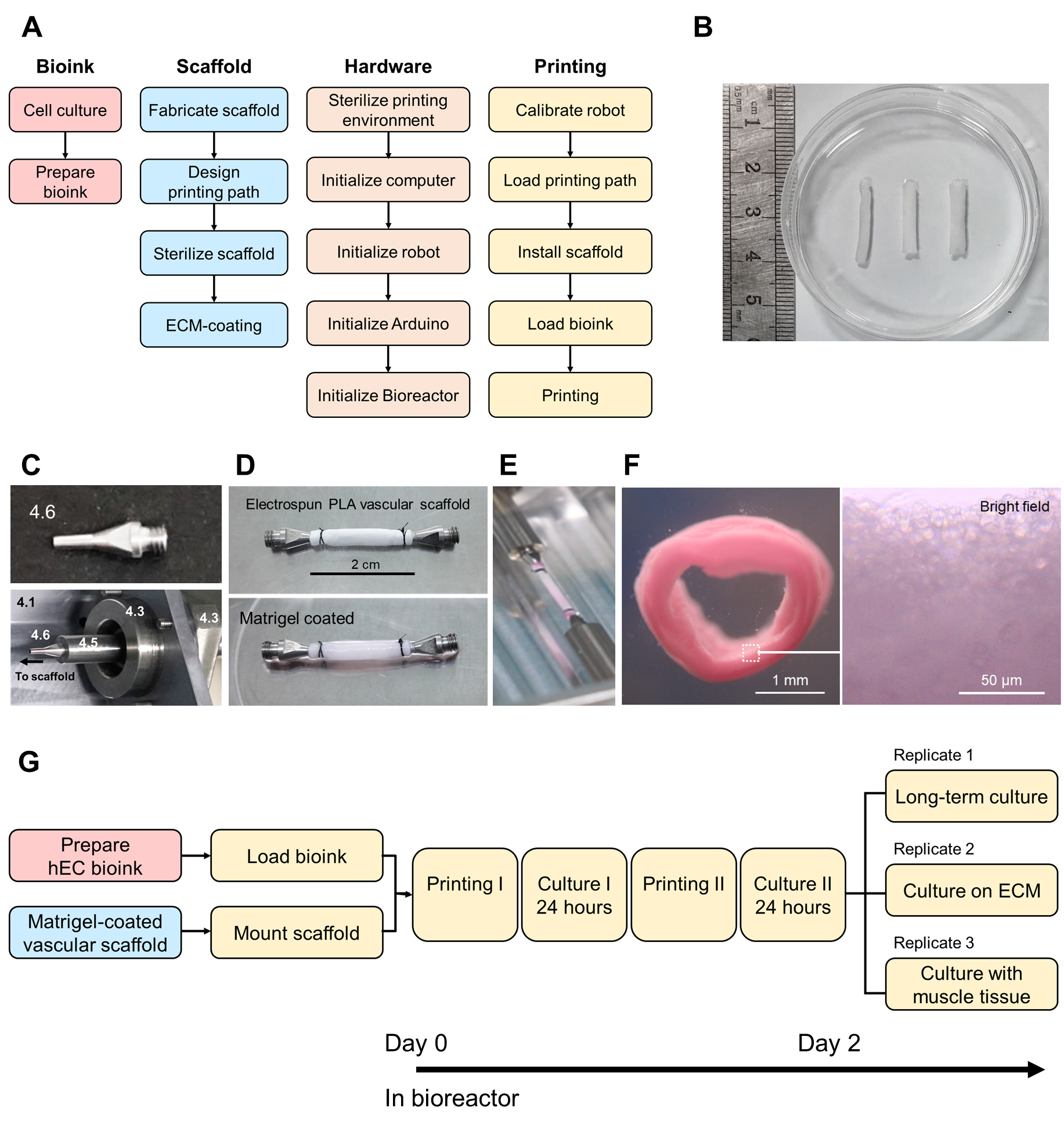


**Fig. S10. A general workflow of the 6-DOF robot printing platform and the strategy of printing artificial blood vessels**.

(**A**) Design of the general workflow for a bioprinting task. (**B** to **E**) Poly L-lactate acid (PLLA) scaffolds (**B**) were connected to hollow nozzles at both ends (**C** and **D**), which are then mounted onto the hollow rods inside the bioreactor (**E**). (**F**) Cross-section of hCMEC/d3 cell-perfused tubular scaffold. (**G**) Workflow of printing artificial blood vessels with 2 print-and-culture cycles.


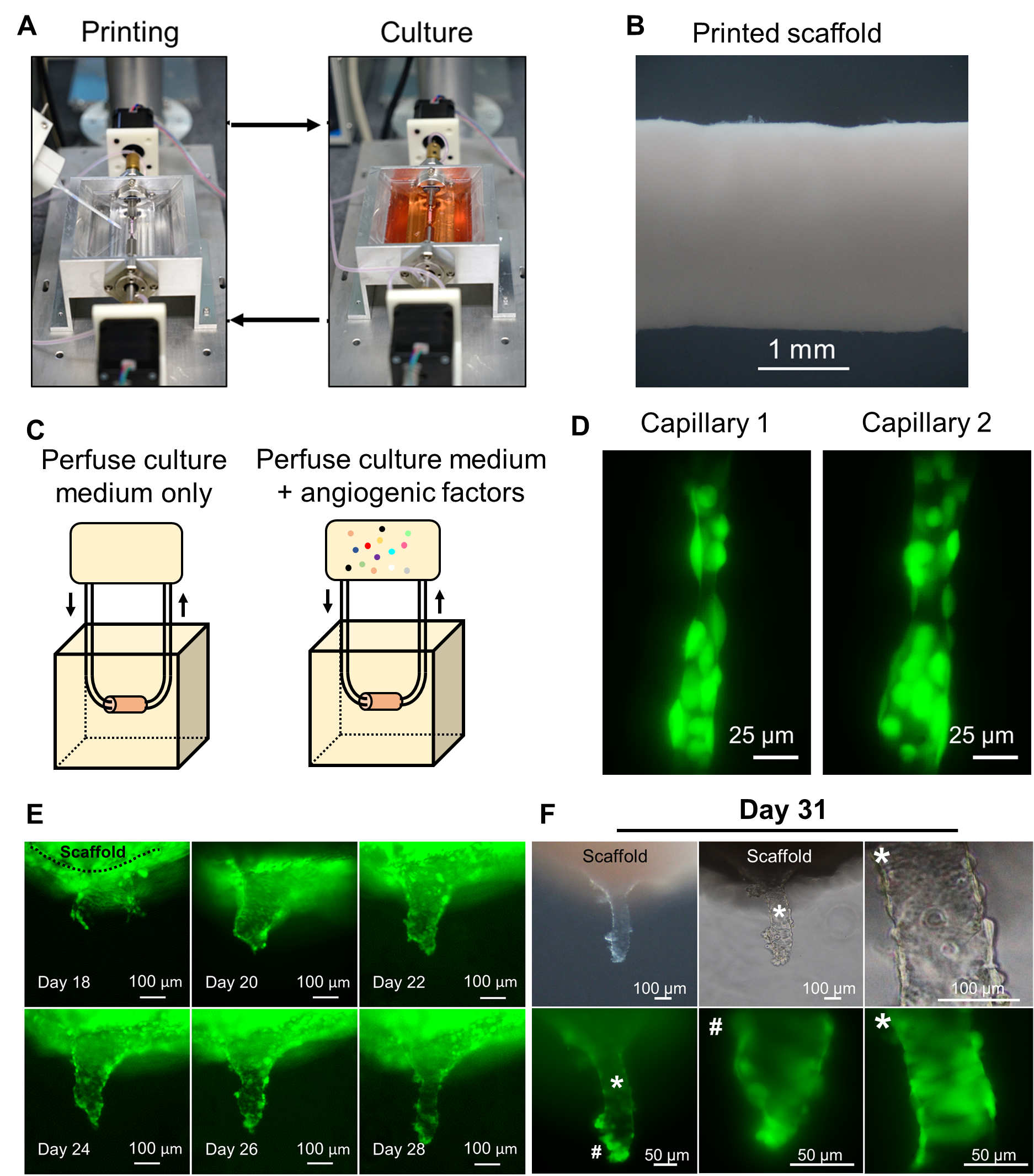


**Fig. S11. Printing and long-term culture of artificial blood vessels.**

(**A**) Setup of the repeated printing-and-culture environment. (**B**) A segment of printed artificial blood vessel. (**C**) Schematic diagram showing the setup for evaluating the effects of adding angiogenic factors (combination of VEGF, FGF, EGF and IGF, see Supplementary Materials and Methods) to the perfusion culture medium. (**D**) Capillary-like structures formed on the artificial blood vessel at post-printing day 13 perfused with angiogenic factors. Shown are two replicates of Fig. 5I. (**E** and **F**) Long-term culture of artificial blood vessels in bioreactor with perfusion of angiogenic factors enables *de novo* formation of actively growing tubular structures. Sprouting outgrowth of the artificial blood vessel shows obvious lumen (marked by *) and endothelial cell-enriched outgrowth tip (marked by #) on day 31. In **D** to **F** , green fluorescent cells are hCMEC/d3-eGFP cells.


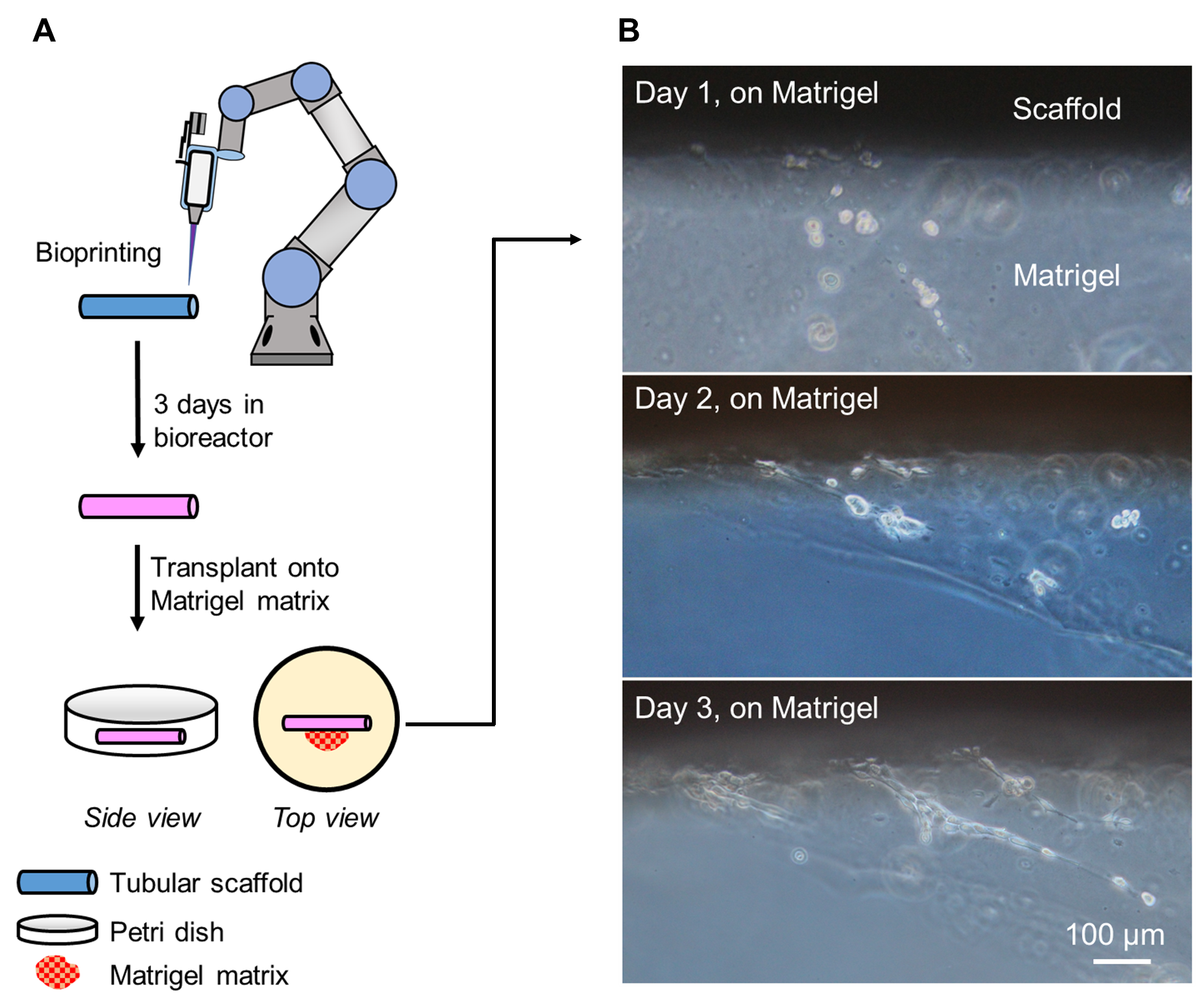


**Fig. S12. Transplantation of artificial blood vessels onto Matrigel matrix induces angiogenic projections.**

(**A**) Schematic diagram showing the workflow of Matrigel-transplantation experiment. (**B**) Artificial blood vessel outgrows capillary-like structures on the Matrigel matrix.

**
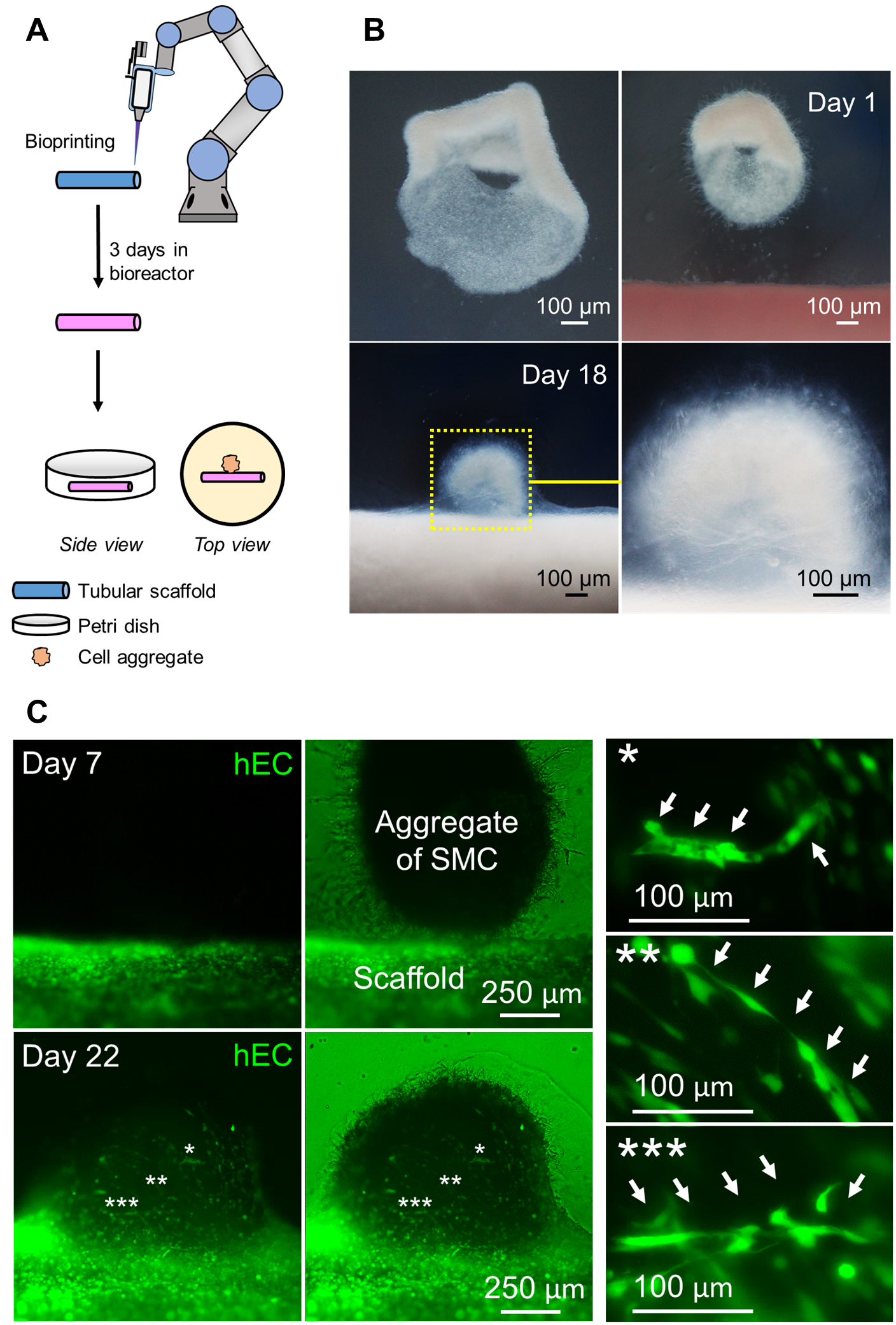
**

**Fig. S13. Joint culture of the artificial blood vessel with an aggregate of smooth muscle cells induces angiogenic projections.**

(**A**) Schematic diagram showing the workflow of the joint culture experiment. (**B**) An aggregate of smooth muscle cells (upper left in **B**) is cultured adjacently to the artificial blood vessel (day 1 upper right in **B**). On day 18, endothelium generated from the artificial blood vessel fused with the smooth muscle cell aggregate. (**C**) Artificial blood vessel forms angiogenic projections into an aggregate of smooth muscle cells (SMC) under joint culture conditions. Left, fluorescence pictures of hCMEC/d3-eGFP cells (green); middle, pictures in left merged with bright field; right, zoom-in of Day 22 images. Arrows indicate capillary-like structures. hEC, hCMEC/d3 cells.


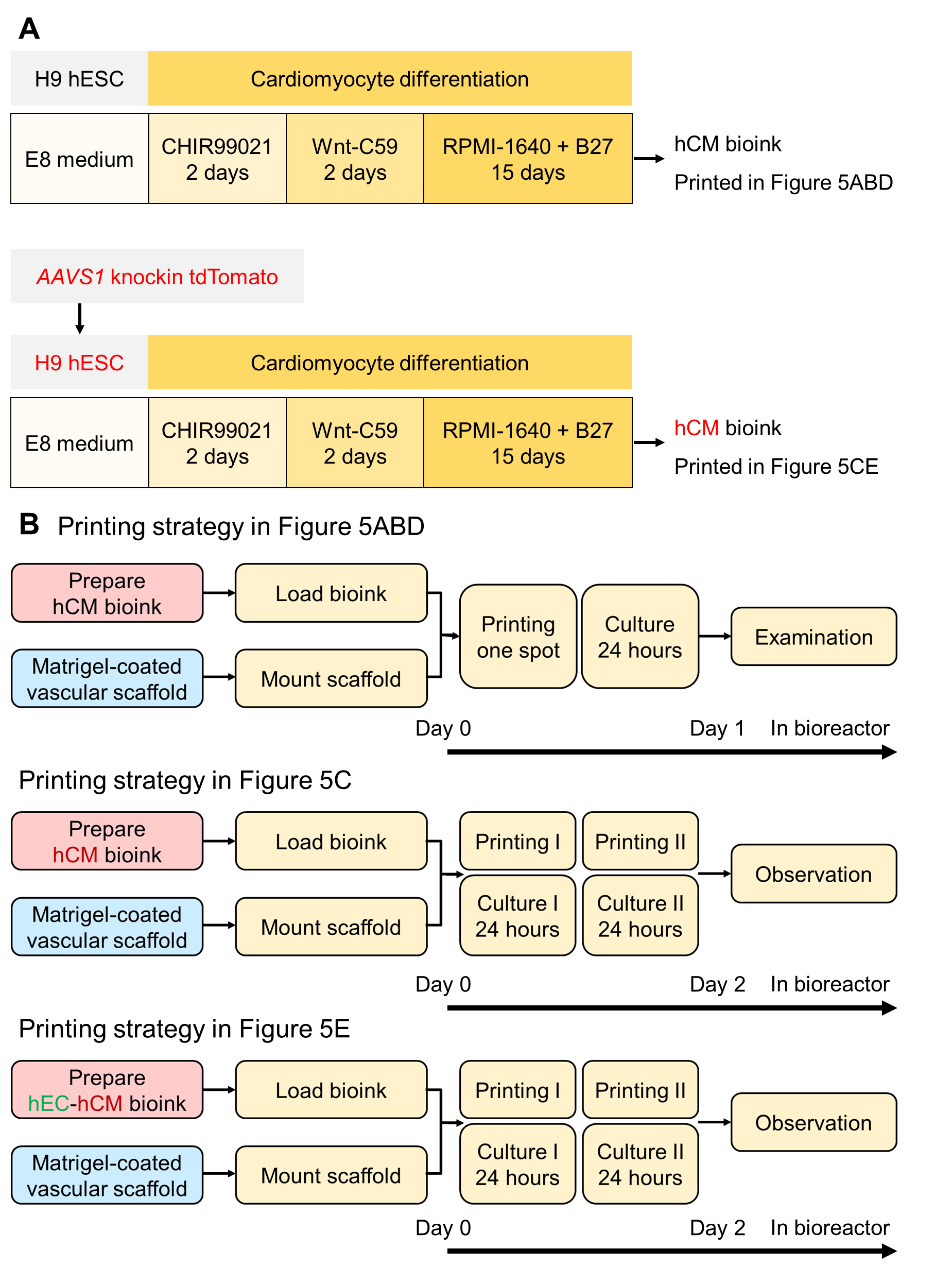


**Fig. S14. Workflows for human cardiomyocyte differentiation and cardiomyocyte bioink preparation and printing.**

(**A**) Workflow for differentiating H9 hESC into human cardiomyocytes (hCM) by modulating Wnt signaling pathway. (**B**) Workflow for printing cardiac tissues in Fig. 5. hEC, hCMEC/d3-eGFP cells.

**
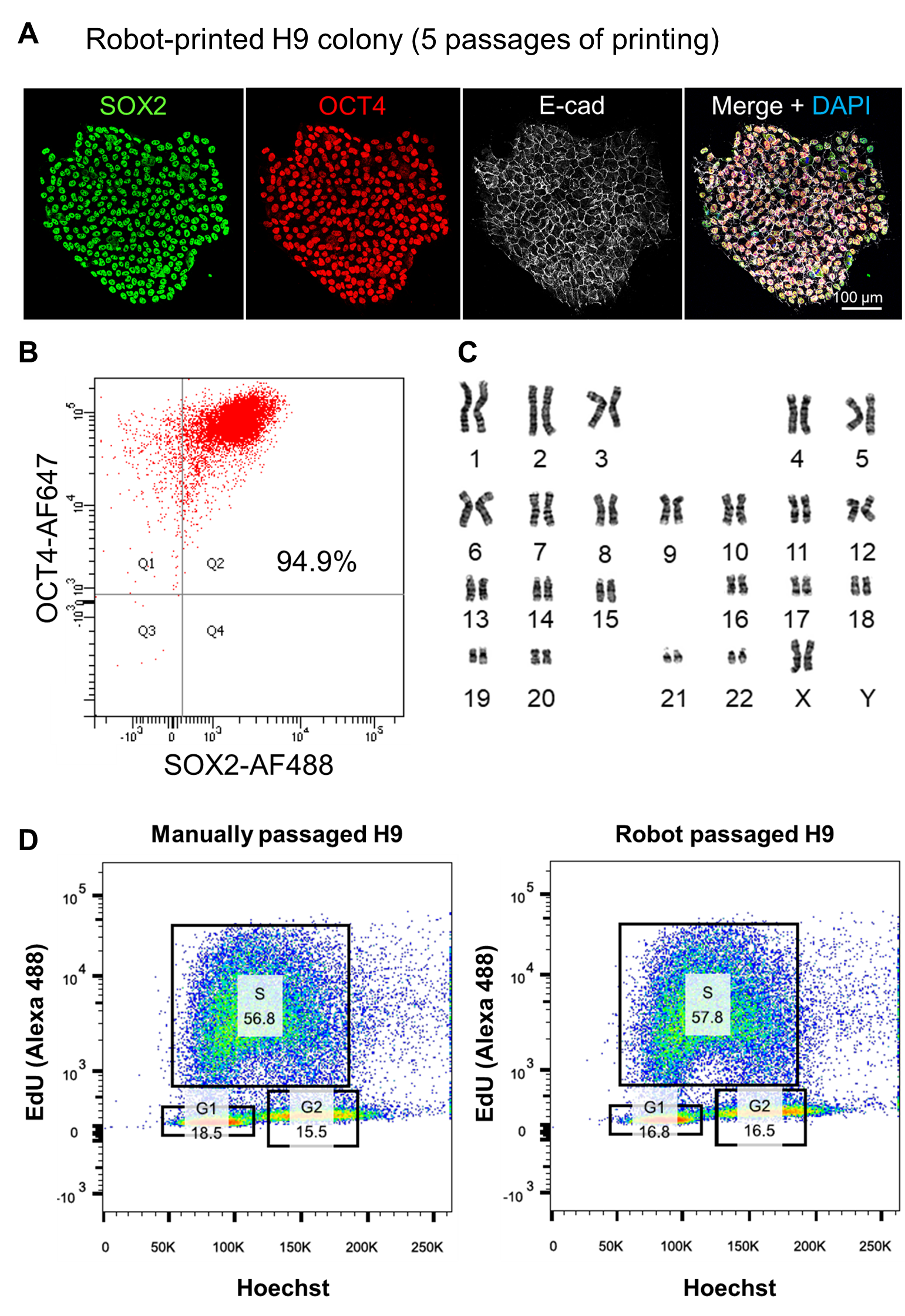
**

**Fig. S15. Robot printed H9 hESCs preserve pluripotency, genome integrity and proliferation ability.**

(**A**) Robot-printed H9 colonies preserve SOX2^+^ and OCT4^+^ features. (**B**) FACS analysis of H9 hESCs for pluripotency assessment (SOX2^+^ and OCT4^+^). (**C**) Robot printed H9 hESCs are karyotyped normal. (**D**) Robot printed H9 hESCs proliferate normally as comparing with the manually passaged ones. In this figure, printed H9 hESCs all refer to H9 hESCs after 5 consecutive passages by robot printing.

**
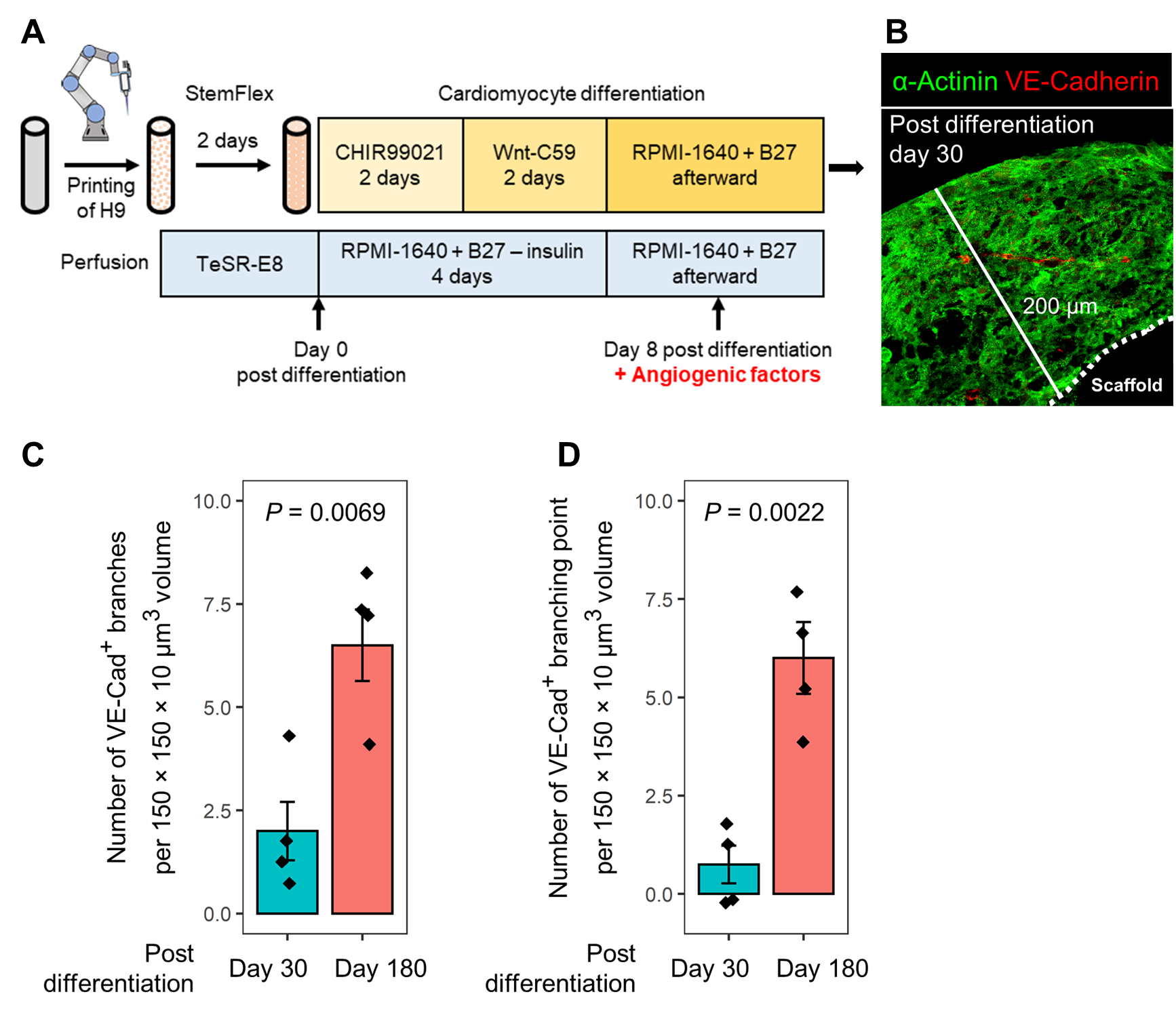
**

**Fig. S16. Long-term culture of print-and-differentiate cardiac tissues in bioreactor generates inter-connected vascular networks.**

**(A)** Schematic workflow of printing and differentiation conditions for H9 hESCs within the bioreactor. (**B**) Visualization of differentiated vascularized cardiac tissue. (**C** and **D**) Statistical comparison of VE-cadherin^+^ tubular branches (**C**) and branching points (**D**) in cardiac tissues at day 30 or day 180 post-differentiation. Error bars are standard error of the mean of 4 replicates. *P*-values are calculated by Student’s *t*-test.


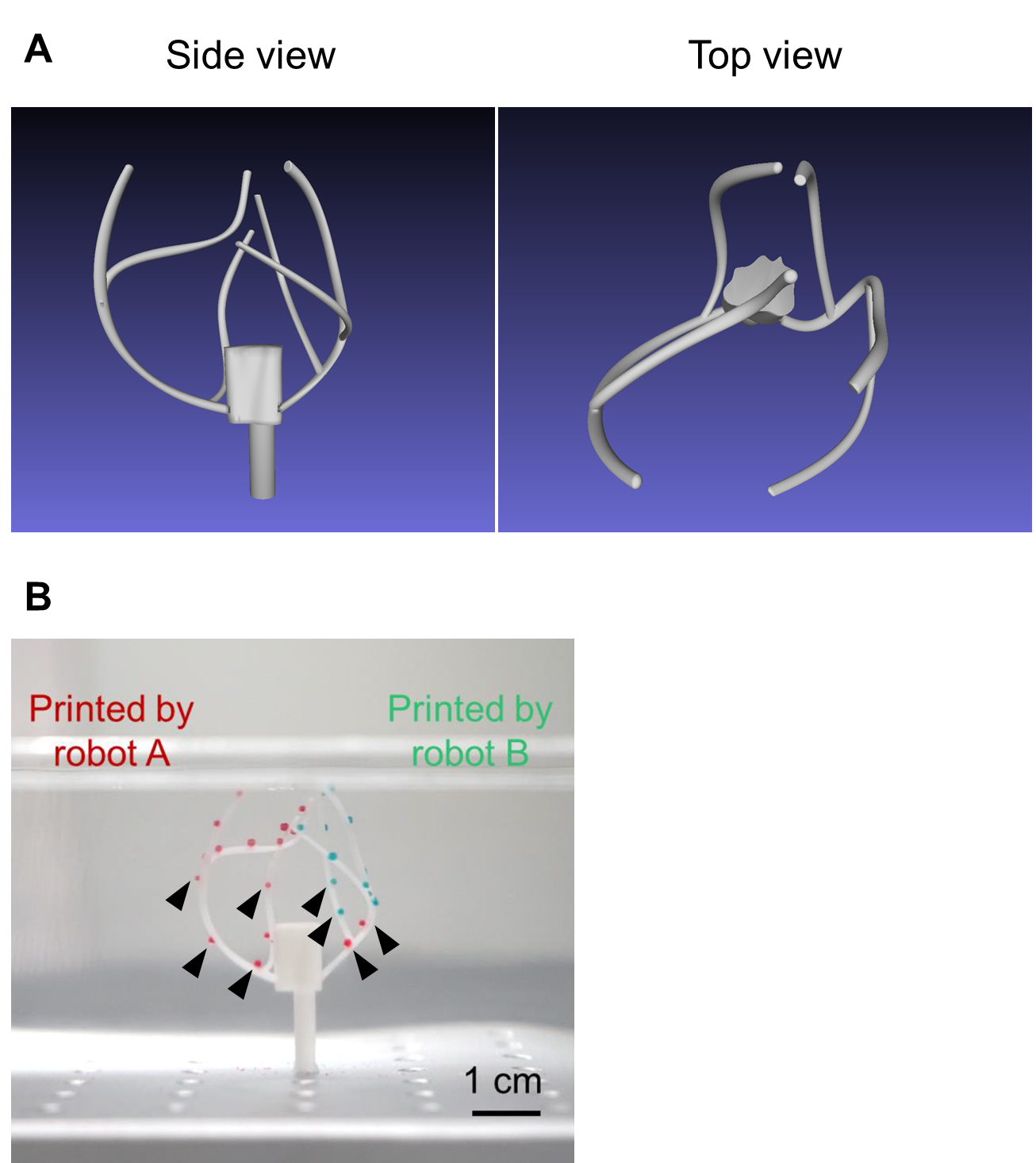


**Fig. S17. Six-DOF robot bioprinter outperforms Cartesian bioprinter in printing bioinks on complex-shaped coronary scaffold.**

(**A**) Three dimensional renderings show the design of complex coronary scaffold. (**B**) Unreachable positions on the coronary scaffold when confining the movement of the six-DOF robot bioprinter in XYZ directions to simulate a Cartesian bioprinter (Compared with Fig. 6).

**Captions for Supplementary Videos**

**Video S1. Setup of the stepper motor-controlled cell ejector on the end-effector of a 6-DOF robot.**

Gentle motion of the stepper motor drives liquid extrusion of the Multipette. See also Fig. S2.

**Video S2. Setup of the electromagnet cell ejector.**

Motion of the electromagnet ejector causes severe vibration of the Multipette.

**Video S3. Recording of the 6-DOF robot movement.**

The 6-DOF robot (UR3) is programmed to move freely to show its superiority in multi-directional bioprinting.

**Video S4. Recording of heart drawing experiment on a human heart model.**

The 6-DOF robot carrying a red marker pen is drawing a coronary network on a plastic 3D human heart model. See also Fig. S5A-C.

**Video S5. Recording of cell printing on a tubular scaffold.**

Shown is the process of cell printing onto the upper 1/3 sector of a tubular scaffold.

**Video S6. Contraction of cardiac layer on post-printing day 10.**

Shown is the live recording of cardiomyocyte contraction on post-printing day 10. Related to Fig. 5C.

**Video S7. Contraction of vascularized cardiac tissue on post-printing day 3.**

Shown is the collective contraction of mix printed cardiomyocytes (red cells) and endothelial cells (green cells) on post-printing day 3. Related to Fig. 5E.

**Video S8. Contraction of cardiac tissue on post-differentiation day 12.**

Shown is the first observable contraction of cardiomyocytes differentiated from printed H9 hESCs on post-differentiation day 12.

**Video S9. Contraction of cardiac tissue on post-differentiation day 180.**

Shown is the collective contraction of cardiomyocytes differentiated from printed H9 hESCs on post-differentiation day 180.

**Video S10. Two-robot platform enables cooperative printing of two bioinks on complex-shaped coronary scaffold.**

Shown is the cooperative printing process of two robot-based bioprinters. Related to Fig. 6A and B.

**Video S11. Two-robot platform enables jointly printing of two bioinks on complex-shaped coronary scaffold.**

Shown is two robot-based bioprinters jointly print two bioinks onto the same branch of a complex-shaped coronary scaffold. Related to Fig. 6A and C.
